# A Reliable and Secure Cluster-Routing Framework for Drone-Assisted Disaster Management in Smart Cities

**DOI:** 10.3390/s26113352

**Published:** 2026-05-25

**Authors:** Bader Alwasel, Ahmed Salim, Pravija Raj Patinjare Veetil, Ahmed M. Khedr, Walid Osamy

**Affiliations:** 1 Unit of Scientific Research, Applied College, Qassim University, Buraydah 52571, Saudi Arabia; 2 Mathematics Department, Zagazig University, Zagazig 44519, Egypt; 3 Computer Science Department, University of Sharjah, Sharjah 27272, United Arab Emirates; 4 Computer Science Department, Faculty of Computers and Artificial Intelligence, Benha University, Benha 13511, Egypt

**Keywords:** disaster management, flying ad hoc networks (FANETs), post-disaster recovery, routing, trust, unmanned aerial vehicles (drones)

## Abstract

Natural and human-made disasters can severely impair terrestrial communication infrastructures and disrupt emergency response coordination in modern smart cities. To address these challenges, this paper introduces the Weighted Average *Yo-Yo*-based Clustering and Routing (WAY-CR) scheme, an adaptive, secure, and energy-efficient drone-assisted solution for post-disaster network recovery and emergency response. WAY-CR integrates three main components: First, a novel WAY-based metaheuristic optimizer incorporates the concept of Yo-Yo Motion into the conventional Weighted Average Algorithm (WAA), improving the balance between exploration and exploitation during CH selection and clustering. Second, a secure communication model combines the Paillier Homomorphic Cryptosystem (PHC) with a trust evaluation model to provide end-to-end security and authenticity, ensuring that only authenticated and trustworthy drones participate in communication and routing. Third, a Trust-Aware Boltzmann Path Selection method introduces probabilistic decision-making into routing, allowing adaptive selection of secure and energy-efficient routing paths. WAY-CR formulates a multi-objective optimization model that minimizes communication cost and energy consumption while maximizing trust, link stability, and coverage. Stage 1 addresses secure intra-Ground Control Station (GCS) clustering, authentication, and trust management, whereas Stage 2 restores inter-GCS connectivity through a Secure Relay Discovery and Verification procedure based on Boltzmann Path Selection. An adaptive maintenance mechanism further supports dynamic reconfiguration in response to CH failures, mobility, or trust degradation, thereby preserving stable network performance under disaster-induced disruptions. Extensive simulation results show that WAY-CR outperforms state-of-the-art Flying Ad Hoc Network (FANET) baselines in energy efficiency, cluster stability, trust accuracy, and end-to-end packet delivery, highlighting its potential as a resilient, scalable, and secure solution for post-disaster smart-city environments.

## 1. Introduction

Natural and human-made disasters pose serious threats to communities, infrastructure, and communication systems. During such events, terrestrial communication facilities, including cellular towers and radio repeaters, may become partially or completely unavailable, leaving first responders without reliable channels for coordination and information exchange [1,2,3,4,5]. This limitation highlights the need for communication solutions that are adaptive, robust, and rapidly deployable in disrupted environments.

In this context, Unmanned Aerial Vehicles (UAVs), also referred to as drones, have emerged as an effective platform for post-disaster communication and emergency support because of their mobility, flexibility, and cost-effectiveness. Their ability to access hazardous or hard-to-reach areas makes them suitable for tasks such as aerial surveillance, victim detection, real-time mapping, and communication restoration [6]. Advances in artificial intelligence and sensing technologies further enhance drone capabilities by supporting autonomous navigation, survivor identification, and real-time data transmission [7,8,9,10]. Drones have also shown effectiveness across the pre-disaster, response, and recovery phases of disaster management, using fixed-wing platforms for large-area monitoring and rotary-wing platforms for localized tactical missions [11]. Equipped with thermal sensors, millimeter-wave radar, and GPS modules, drones can detect survivors, navigate complex environments, and transmit situational data in real time [12].

To extend the capabilities of individual drones, *Flying Ad Hoc Networks (FANETs)* have attracted increasing attention as a multi-drone communication paradigm that operates without fixed infrastructure. FANETs enable rapid deployment, collaborative coverage, and resilient communication in disaster environments [13,14,15]. However, their design remains challenging because clustering and routing must operate reliably under high mobility, energy limitations, dynamic topology changes, and potential security threats. Addressing these challenges is essential for maintaining network survivability and secure data exchange during rescue and recovery operations.

### 1.1. Background and Motivation

Research on drone-assisted disaster response can be broadly viewed from two complementary perspectives. The first focuses on system-level emergency communication, where drones are used to restore connectivity, extend coverage, and support secure backhaul links. The second focuses on network-layer clustering and routing in FANETs, where self-organization and data forwarding must be maintained despite mobility, resource constraints, and adversarial conditions. Although substantial progress has been made in both directions, most existing studies address connectivity, efficiency, or security separately rather than within an integrated and adaptive FANET framework.

At the system level, prior studies have demonstrated the feasibility of drone-assisted emergency communications in disaster scenarios. Examples include temporary ad hoc networking for victims and responders under bandwidth and flight-time constraints [16], resource allocation for drone-mounted base stations after terrestrial infrastructure failure [17], and optimized drone placement, trajectory planning, and user clustering in software-defined networking (SDN)-based architectures to improve throughput, energy efficiency, and convergence speed [18,19]. Drone-based sensing has also been used for infrastructure damage assessment and situational awareness, including search-and-rescue human detection and environmental disaster monitoring through joint aerial-ground observation systems [20,21,22]. In addition, tethered drones, distributed clustering of ground devices, free-space optical (FSO) aerial relays, and software-defined multi-drone systems have been investigated to improve coverage continuity, mission duration, and communication responsiveness [23,24,25,26]. Field experiments further confirm that drone-mounted radio repeaters can restore communications within minutes, even in complex terrain [27].

Recent studies have also examined route optimization and adaptive networking for disaster scenarios. Centralized and hierarchical SDN-based routing schemes improve throughput and responsiveness in affected regions [18,26], while mesh-based systems such as UbiQNet support rapid deployment and victim connectivity using commercial drones [28]. Learning-based optimization and NOMA-enabled relay drones have additionally been proposed to enhance scalability and spectral efficiency [29]. Recent work on learning-based communication design has shown that multi-agent reinforcement learning can optimize MAC protocols effectively; however, many such approaches still depend on centralized training, simplified simulation assumptions, and limited generalization, which can reduce practical deployability in highly dynamic environments [30]. Energy-aware path planning, vehicle-routing formulations, low-complexity data collection algorithms, and cooperative drone replacement strategies have also been introduced to cope with limited battery capacity and continuous mission demands [31,32,33]. Despite these advances, many of these frameworks do not explicitly integrate secure data exchange, trust evaluation, and adaptive reconfiguration under adversarial conditions.

To improve resilience and security, several studies have explored blockchain-based and learning-driven control mechanisms. Blockchain-enabled drone networks can strengthen privacy, decentralized coordination, and resistance to cyberattacks in large-scale multi-drone deployments [34], while multi-agent reinforcement learning frameworks support energy-aware, coverage-aware, and context-aware trajectory optimization in disaster scenarios [35]. At the physical layer, FSO communication has been investigated as a high-speed, directional, and low-interception backhaul option for drone-assisted networks when terrestrial infrastructure is unavailable [36]. More broadly, system-level studies on disaster-resilient architectures emphasize the importance of resource allocation, edge intelligence, secure communication, and dynamic network configuration across integrated terrestrial, aerial, and spaceborne systems [37]. Similarly, AIoT-based early-warning platforms show that secure sensing, edge computing, and real-time data fusion can improve disaster response performance [38]. Nevertheless, a unified FANET architecture that jointly incorporates trust management, secure authentication, clustering, routing, and adaptive maintenance remains insufficiently explored.

Motivated by these limitations, this paper proposes the Weighted Average *Yo-Yo*-based Clustering and Routing (WAY-CR) framework, an adaptive, secure, and energy-efficient drone-assisted communication architecture for post-disaster network recovery and emergency response. WAY-CR combines a novel WAY-based metaheuristic optimizer for cluster-head (CH) selection and shelter location planning, a trust-authentication model to exclude unverified drones, a multi-objective fitness function for clustering and routing, and an adaptive maintenance mechanism for dynamic cluster and route reconfiguration.

The framework operates in two stages. In Stage 1, authenticated drones are organized into clusters through a metaheuristic-driven process in which selected CHs manage local communication and data aggregation under a Trust-Aware Routing (TAR) mechanism. An adaptive maintenance strategy preserves cluster stability as network conditions change. In Stage 2, selected CHs act as secure relays to restore connectivity among isolated Ground Control Stations (GCSs) through multi-hop aerial links, thereby enabling mission coordination to resume when terrestrial infrastructure is unavailable.

### 1.2. Major Contribution

**Figure 1:**
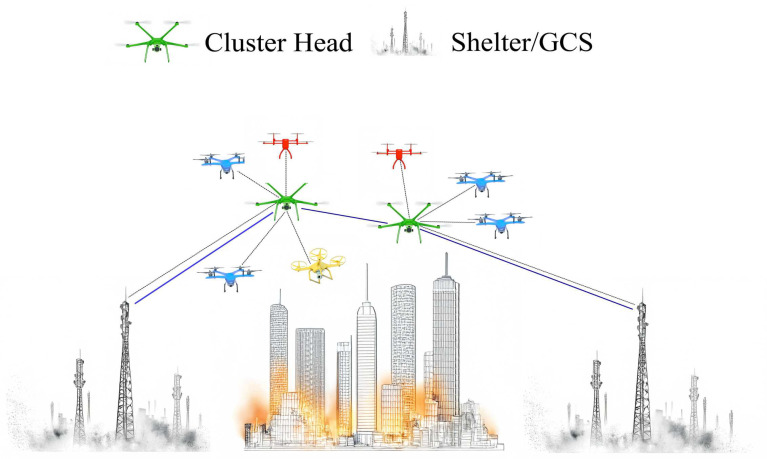
Illustration of the WAY-CR framework for post-disaster communication (fire disaster scenario). Drones form authenticated clusters under each GCS, with selected CHs establishing secure airborne relay links to restore inter-GCS connectivity when terrestrial infrastructure is unavailable.

WAY-CR advances the design of a resilient, scalable, and secure drone-based FANET architecture that can operate effectively in post-disaster environments. By jointly considering optimization, trust, authentication, and dynamic network conditions, the framework contributes to next-generation emergency communication systems capable of saving lives and supporting critical infrastructure recovery. An illustration of the proposed WAY-CR framework in a fire disaster scenario is shown in Figure 1, where the drones form authenticated clusters under each GCS, with selected CHs establishing secure airborne relay links to restore inter-GCS connectivity when terrestrial infrastructure is unavailable. The major contributions of this paper include:

1. A novel WAY-CR framework for secure, efficient, and adaptive drone-assisted communication in post-disaster FANET environments. It integrates optimization intelligence, security mechanisms, and Trust-Aware Routing within a unified architecture.
2. An efficient WAY-based metaheuristic optimizer for intelligent CH selection. By embedding the Yo-Yo Motion Operator into the original Weighted Average Algorithm (WAA), the proposed WAY optimizer effectively balances exploration and exploitation, enhancing clustering stability and coverage efficiency.
3. A unified multi-criteria fitness function for FANET CH selection, formulated as a single, minimizable objective that combines residual energy, intra- and inter-cluster distances, load balance (via Jain’s fairness index), mobility stability, and trust, enabling robust clustering decisions in dynamic drone networks.
4. A Trust-Aware Boltzmann Path Selection mechanism for both intra- and inter-GCS routing stages. By probabilistically selecting best forwarding paths using a composite fitness function in terms of trust, residual energy, link stability, and mobility, it ensures routing reliability, load balancing, and adaptability under frequent topology changes.
5. An adaptive maintenance mechanism to handle CH mobility, trust degradation, and drone failures through dynamic reconfiguration and cluster member (CM) reassignment, thereby maintaining continuous network connectivity while reducing re-clustering overhead during network disruptions.
6. Comprehensive simulations validating the performance of the proposed WAY-CR framework against state-of-the-art FANET clustering and routing protocols. Results demonstrate significant improvements in energy efficiency, cluster stability, trust, and end-to-end packet delivery, confirming its effectiveness in building a resilient and secure drone communication infrastructure for post-disaster scenarios.

The remainder of this paper is organized as follows. Section 2 reviews the related work. Section 3 describes the system model, including network setup, energy consumption, communication, trust computation, and security aspects. Section 4 presents the problem formulation. Section 5 introduces the proposed WAY-CR framework, where it discusses the proposed WAY optimizer algorithm, intra-GCS operations, and inter-GCS communication and relay mechanisms in detail. Section 6 provides the simulation results and performance analysis. Finally, Section 7 concludes the paper and outlines future research directions.

## 2. Related Work

Drone-assisted emergency communications aim to enable rapid recovery, broad coverage, resilience, and secure data exchange. These objectives closely align with the FANET design, where clustering governs drone self-organization and stable CH election, and routing ensures reliable, low-latency, and trustworthy data delivery. Thus, energy efficiency, mobility awareness, scalability, and trust management have emerged as major challenges in FANET, especially in critical post-disaster scenarios. A wide range of the literature has explored clustering and routing strategies tailored for FANETs to improve resilience, scalability, and survivability [39,40,41]. This section critically examines the key advances in clustering and routing mechanisms relevant to our WAY-CR framework. Classical routing baselines have also been examined in FANET environments. Leonov and Litvinov [42] compared the reactive AODV and proactive OLSR protocols in air-to-air mini-drone communications and showed that routing performance is strongly dependent on the selected metric and operating scenario. In their study, AODV achieved better packet delivery and throughput, whereas OLSR provided lower delay and jitter. This comparison highlights an important point for FANET design: routing effectiveness cannot be judged by a single metric, especially under highly mobile aerial conditions. Trust management plays a central role in FANET security. Numerous studies emphasize the significance of trust computation as a foundation for secure clustering and routing and highlight the necessity of adaptive trust evaluation for detecting misbehaving drones and maintaining network reliability [43,44]. A trust-based leader selection mechanism for FANETs (TDLS-FANET) is proposed in [15], incorporating QoS metrics, social trust, and fitness functions to dynamically elect reliable CHs. A fuzzy-logic-based model that blends geocasting with trust-aware node behavior analysis is developed in [45]. In addition, the SCFS scheme [46] employs fuzzy trust evaluation at both the cluster and individual node levels, permitting nodes with multiple opportunities to prove their trustworthiness before final classification, thereby enhancing overall network security. This study also introduces a Cluster Confidence (C) metric to strengthen routing security and overall network trustworthiness. The Fuzzy-Based Trusted Malicious Drone Detection Scheme proposed in [47] enhances the security and reliability of FANETs. It employs a fuzzy logic-based trust evaluation mechanism that integrates multiple parameters, enabling a more accurate distinction between intentional and accidental misbehavior, supporting secure clustering. By incorporating a dynamic trust decay function, the system continuously adapts to network changes and drone behavior over time. To further strengthen authentication and trust propagation, blockchain-based strategies have been explored. The Trust Establishment Mechanism for FANET (TEM-FANET) in [48] presented a lightweight blockchain-based trust and authentication system designed to identify malicious drones in real time, while [49] extended this concept by developing a hybrid FANET trust model incorporating flying and stationary nodes, using a blockchain-based record system to securely share and manage trust information among network nodes.

Beyond trust-centric designs, several works employ metaheuristic optimization and learning techniques to address FANET clustering and routing challenges. The CLARA technique in [50] is a five-phase routing algorithm that uses learning automata to adapt CH selection and form routes based on energy and link quality. A combination of game theory and decision trees with path similarity is employed in [51] to form stable clusters. The *k*-means clustering is employed in [52]; however, the fixed selection of the *k* value limits its effectiveness, leading to a significantly shorter cluster lifetime. A mobility-aware clustering scheme, MWCRSF, introduced in [53], is based on the Sparrow Search Algorithm (SSA), where the CH selection is governed by a weighted function over mobility, connectivity, energy, and distance. Hybrid and bio-inspired routing approaches have also gained attention. The ICRP method [14], a hybrid ant colony routing protocol enhanced by Physarum-inspired behavior, dynamically adjusts routes using predictive maintenance and pheromone feedback. Bio-inspired clustering methods have been explored using Grey Wolf Optimization (GWO) and Moth Flame Optimization (MFO), targeting energy-efficient clustering [54,55]. The GWO-based clustering for wildfire monitoring in [54] reduces energy consumption and minimizes routing delays. The MFO-based method in [55] considers drone location and residual energy during CH election. However, these methods often incur high clustering overhead, neglect Cluster Maintenance, or fail to account for drone mobility and trust, limiting their reliability and applicability in dynamic environments. Latency and energy efficiency remain critical constraints for FANETs. A latency-oriented clustering scheme using reward indices, distance, and speed is presented in [56]. The ICBM-UAV technique [57] focuses on search-and-rescue missions, forming intelligent clusters for victim detection while minimizing delay and energy usage. HMGOC [58] combines Mountain Gazelle Optimization (MGO) with the Jaya algorithm for clustering and uses Bayesian inference for routing decisions, improving stability and latency performance. Scalability concerns have driven the adoption of biologically inspired models. The PICA framework [59], inspired by Physarum behavior, improves CH stability and cluster merging while minimizing reclustering using distributed multi-hop logic.

Recently, optimization and trust-integrated methods have been gaining popularity. TMFCS [60] integrates trust evaluation with fitness-based clustering and introduces a maintenance phase to limit reclustering frequency. The Squirrel Search Optimization-based approach (SSAFANET) in [41] jointly considers residual energy, trust, mobility, node degree, and distance to the ground station, with CH reselection rules for cluster stability. Routing is performed using a multi-hop cost function based on hop distance and residual energy. The ICW technique in [40] employs a Whale Optimization Algorithm (WOA)-based method to form mobility-aware clusters with adaptive hello intervals. CHs are selected based on a score combining residual energy, average link lifetime, neighbor degree, and average neighbor distance. It employs intra-cluster greedy routing to the CH and inter-cluster multi-hop CH-to-CH routing, where next hops are chosen by ranking residual energy, distance to destination, and link lifespan. SSACEER [61] targets disaster monitoring scenarios such as forest fire using SSA-based clustering that selects CHs via a weighted sum of residual energy, distance to the BS, relative mobility, and degree difference, and position-based greedy routing, where the next hop is chosen according to link lifetime and the future direction of one-hop neighbors to ensure reliable delivery to the BS. The Adaptive Artificial Fish Swarm Strategy (AAFSA) [62] integrates trust-aware CH selection with ITOPSIS-based multi-hop routing that ranks candidate routes using metrics such as flight autonomy, trust value, mobility, neighbor range, link quality, and RSSI to choose reliable paths. More recently, EESCRA [63] introduced a trust- and energy-aware clustered routing approach, where drones are modeled on a bipartite graph. The malicious drones are excluded based on a threshold, and routes are ranked based on a composite metric that jointly considers average path trust and energy costs.

Overall, recent works show a growing trend toward integrating trust-aware, adaptive, and bio-inspired techniques in FANET routing. However, most approaches focus on isolated aspects, such as clustering stability, routing efficiency, or trust management, without holistically integrating optimization, trust verification, routing adaptability, and maintenance within a unified framework. Moreover, many schemes lack explicit mechanisms to jointly address mobility dynamics, malicious behavior, and post-disaster operational constraints. The proposed WAY-CR framework aims to bridge this gap by combining multi-objective optimization, trust verification, and adaptive routing maintenance to meet the demands of dynamic post-disaster response environments. Table 1 gives a comparison of trust-aware and optimization-based clustering and routing methods in FANETs.

## 3. System Model

This section presents the system architecture and the foundational assumptions supporting the proposed framework, including the network and communication energy modeling, mobility characteristics, and trust and security model employed in this work.

### 3.1. Network Model

The network comprises *N* drones distributed in a 3D space, represented as an undirected graph $G_{F}=\left(V_{F},E_{F}\right)$, where $V_{F}$ denotes the set of drones, and $E_{F}$ represents the communication links between them. The drones follow a smooth 3D random-walk model (SmoothRW3D) [64]. Each drone is equipped with location-aware sensors (e.g., GPS, IMU) and wireless communication interfaces. The drones are dynamically organized into clusters consisting of a CH and several CMs. CHs are responsible for: (i) monitoring and managing their respective clusters, (ii) coordinating communication with the shelters or GCSs through CHs, (iii) enabling both intra-cluster and inter-cluster communication, and (iv) periodically updating and maintaining the list of active CMs. CMs perform sensing and transmission tasks under the supervision of the CH.

**Assumptions:**  The following assumptions are considered in this work:

1. Each drone *U* has a unique identifier ($U_{ID}$).
2. Drones are mobile, and the distances between them change over time.
3. Drones are equipped with wireless transceivers and location sensors (e.g., GPS, IMU).
4. The communication range of each drone is denoted as *R*.
5. Each drone knows its current position $\left(U_{x},U_{y},U_{z}\right)$ and velocity $\left(U_{vx},U_{vy},U_{vz}\right)$.
6. Two drones $\left(U_{1},U_{2}\right)$ are neighbors if the Euclidean distance between them, given by $Dist_{U_{1}U_{2}}={\left({\left(U_{1x}-U_{2x}\right)}^{2}+{\left(U_{1y}-U_{2y}\right)}^{2}+{\left(U_{1z}-U_{2z}\right)}^{2}\right)}^{1/2}$, is less than *R*. If this condition is satisfied, the pair $\left\{U_{1},U_{2}\right\}$ forms an edge in $E_{F}$.

Similar to many existing drone/FANET studies considered for comparative analysis [15,39,40,41,60,61,62,63], this work assumes the availability of sufficiently accurate local information, such as position, neighbor connectivity, and trust-related observations for decision-making. This assumption is adopted to maintain the tractability of the proposed framework in terms of design and evaluation. In practical deployments, however, such information may be affected by sensing inaccuracies, communication delays, intermittent links, and incomplete observations.

### 3.2. Communication Energy Model

The drones consume energy primarily during transmission, reception, hovering, and flying. The data transmission energy is modeled using the first-order radio model [65]. The energy $E_{TR}\left(j,dis\right)$ to transmit *j* bits over distance dis and to receive $E_{RC}\left(j\right)$ a *j*-bit message are given as follows:

(1) $$E_{TR}\left(j,dis\right)=\left\{\begin{array}{cc} jE_{ele}+j\epsilon_{fs}dis^{2} & \mathrm{if}\;dis<dt\\ jE_{ele}+j\epsilon_{amp}dis^{4} & \mathrm{otherwise} \end{array}\right.\;\mathrm{and}\;E_{RC}\left(j\right)=jE_{ele}$$

where $E_{ele}$: energy for processing per bit, $\epsilon_{fs}$, $\epsilon_{amp}$: amplifier parameters, and dt: threshold distance, respectively.

### 3.3. Flight Energy Model

Hovering and flying power are calculated as in [65,66]:

(2) $$P_{U_{h}}=\sqrt{\frac{{\left(m_{U}\cdot g\right)}^{3}}{2\pi w_{r}^{2}w_{n}\rho_{a}}},\;\;\;\;P_{U_{f}}=\left(P_{max}-P_{U_{h}}\right)\left(\frac{v_{U}\left(t\right)}{v_{max}}\right)$$

where $P_{U_{h}}$ and $P_{U_{f}}$ denote the drone hovering power and flying power, and $P_{max}$ is the maximum power, respectively; $P_{\max}$ is the maximum drone power; $m_{U}$ is the drone mass; *g* is the gravitational acceleration; $w_{n}$ and $w_{r}$ are the number and radius of the rotors, respectively; $\rho_{a}$ is the air density; $v_{U}\left(t\right)$ is the drone speed at time *t*; and $v_{\max}$ is the maximum drone speed.

The total energy consumed ($E_{U_{h}}$) during hovering and flying is:

(3) $$E_{U_{h}}=P_{U_{h}}\cdot t_{h},\;\;\;\;E_{U_{f}}=\int_{0}^{t_{f}}P_{U_{f}}\;dt$$

where $t_{h}$ and $t_{f}$ are the durations of hovering and flying.

### 3.4. Trust Model

This section describes an adapted trust model designed specifically for FANETs, where highly mobile drones dynamically form a wireless communication network. The model extends the conventional WSN trust mechanism [67] by incorporating mobility-aware metrics, dynamic clustering, and aerial-specific constraints. In each communication round, drones exchange data and monitor neighboring drones’ behavior. Trust is evaluated based on first-hand observations and periodically exchanged reports. Trust computation occurs in two tiers: at the Drone tier and the GCS or Distributed tier, as follows.

#### 3.4.1. Drone Tier Trust Computation

Each drone computes a first-hand trust value for neighboring drones based on a weighted combination of mobility-aware metrics, such as packet forwarding ratio, signal strength, link duration, position consistency (GPS accuracy), and mobility deviation (flight path stability). These metrics are physically interpretable in FANET operation: packet forwarding ratio reflects relay cooperation, signal strength and link duration capture wireless link stability, position consistency indicates navigation plausibility, and mobility deviation characterizes flight-path stability. The trust value of drone *i* as observed by drone *j* is calculated using:

(4) $$FH\left(j,i\right)=\sum_{k=1}^{l}w_{k}\cdot t_{k}\left(j,i\right)$$

where $t_{k}\left(j,i\right)$ is the score of metric *k* for drone *i* as observed by drone *j*, $w_{k}$ is the weight assigned to metric *k*, and $\sum_{k=1}^{l}w_{k}=1$. Due to node mobility, trust values are decayed over time when no recent observations are available, ensuring outdated information is gradually disregarded.

#### 3.4.2. Cluster-Based Aggregation and Trust Propagation

Drones periodically form dynamic clusters. Within each cluster, the cluster members (CMs) send trust assessments of peer drones to the cluster head (CH), which aggregates these reports according to $CT\left(i\right)=\frac{1}{b}\sum_{r=1}^{b}FH\left(r,i\right),$ where CT(i) denotes the cluster-level trust value of drone *i*, *b* is the number of CMs that reported on drone *i*, and FH(r,i) is the first-hand trust value assigned by node *r* to node *i*.

To compute the overall trust of a drone, we employ an Exponentially Weighted Moving Average (EWMA) model, which smoothly blends new trust evidence with historical values. This gives more importance to recent behavior while still retaining the past trust context:

(5) $$OT{\left(i\right)}_{t}=\lambda \cdot \left(w_{a}\cdot FH{\left(CH,i\right)}_{t}+w_{b}\cdot CT{\left(i\right)}_{t}\right)+\left(1-\lambda \right)\cdot OT{\left(i\right)}_{t-1}$$

where $OT{\left(i\right)}_{t}$ is the updated overall trust of drone *i* at time *t*, $FH{\left(CH,i\right)}_{t}$ is the CH’s direct observation of node *i*, $CT{\left(i\right)}_{t}$ is the aggregated collective trust from other CMs, λ∈(0,1) is the smoothing factor that determines the influence of new vs. past information, $w_{a}+w_{b}=1$ are source weighting parameters, allowing emphasis control between direct and indirect trust. The EWMA-based formulation provides a resilient trust estimation mechanism that dynamically adapts to drone behavior over time, even under network volatility.

When connected to a GCS, trust reports are aggregated for centralized evaluation; otherwise, drones perform a distributed trust assessment through a CH. The GCS or the CH compares computed trust values against predefined thresholds, excluding nodes that fall below these limits from routing or cooperative tasks. Validated data increases a drone’s trust score, thereby enhancing overall network reliability.

### 3.5. Security Model

The security model employed in the WAY-CR framework uses homomorphic encryption to enable secure *drone-to-drone* and *drone-to-GCS* communication through public-key cryptography, while still allowing computations to be performed directly on encrypted data. This ensures that sensitive information remains protected even in untrusted environments. Among existing schemes, the Paillier cryptosystem [68] is widely adopted because of its additive homomorphic property, which allows encrypted values to be combined without decryption. Paillier-based homomorphic encryption has also been successfully applied in drone-based systems. For instance, Yan et al. [69] proposed a secure distributed estimation method that uses partially homomorphic encryption to protect transmitted measurements and estimates. Similarly, Alzahrani et al. [70] employed a homomorphic encryption method for secure communication and proposed a key regeneration algorithm based on Paillier homomorphic encryption.

The security of the Paillier cryptosystem relies on the hardness of the composite residuosity class problem. If two messages are encrypted under the same public key, the product of their ciphertexts decrypts to the sum of the corresponding plaintexts modulo *n*. Thus, the Paillier cryptosystem satisfies the following homomorphic property:

(6) $$D\;\left(E\left(m_{1}\right)\cdot E\left(m_{2}\right)\;\mathrm{mod}\;n^{2}\right)=m_{1}+m_{2}\;\left(\mathrm{mod}\;n\right).$$

Let $c=E\left(m_{1}\right)\cdot E\left(m_{2}\right)\;\mathrm{mod}\;n^{2}.$ Then,

(7) $$c=\left(g^{m_{1}}r_{1}^{n}\;\mathrm{mod}\;n^{2}\right)\left(g^{m_{2}}r_{2}^{n}\;\mathrm{mod}\;n^{2}\right)\;\mathrm{mod}\;n^{2}\;=g^{m_{1}+m_{2}}{\left(r_{1}r_{2}\right)}^{n}\;\mathrm{mod}\;n^{2}.$$

Therefore, $D\left(c\right)=m_{1}+m_{2}\;\left(\mathrm{mod}\;n\right).$ Here, E(·) and D(·) denote the encryption and decryption functions, respectively, in the Paillier cryptosystem. The parameter n=pq, where *p* and *q* are two large prime numbers, is part of the public key and defines the plaintext space $Z_{n}$. The value *g* is another public parameter, chosen such that the decryption procedure is valid; in the standard Paillier construction, a common choice is g=n+1. Thus, a message $m\in Z_{n}$ is encrypted as $E\left(m\right)=g^{m}r^{n}\;\mathrm{mod}\;n^{2},$ where $r\in Z_{n}^{*}$ is a random value, and decryption recovers the original message by applying D(·) to the ciphertext.

The proposed security model is integrated across all stages of the WAY-CR framework. During the *Drone Registration* and *Cluster Formation* stages, homomorphic encryption is used to enable privacy-preserving authentication and membership verification by allowing computations on encrypted data without revealing the underlying information, thereby helping prevent unauthorized drone participation. In the *Trust-Aware Routing*, *Cluster Maintenance*, and *Inter-GCS Communication* phases, encrypted trust metrics and routing information enable secure computation and information exchange without exposing sensitive data, thereby supporting confidentiality and privacy preservation across the network.

#### Compromised Node Attack Model

We consider an insider adversary scenario in which a drone or CH, after successful registration, becomes compromised due to capture, malware injection, or key exposure. A compromised node is, therefore, a legitimate network participant with valid protocol access, but with malicious behavior. Such a node may: (i) advertise false state information (e.g., energy, location, or trust-related reports), (ii) selectively drop, delay, or misroute packets, (iii) attempt to attract traffic by presenting itself as a favorable relay/CH, and (iv) replay or inject previously observed control packets.

However, the adversary is assumed not to break the underlying cryptographic primitives, forge valid GCS signatures, or compromise all GCSs simultaneously. Under this model, WAY-CR limits the impact of compromised nodes through authenticated registration, signature-based inter-GCS relay verification, Trust-Aware Routing that penalizes unreliable forwarding behavior and Adaptive Cluster Maintenance that triggers re-association or CH replacement when abnormal activity is detected.

## 4. Problem Formulation

After a large-scale disaster (e.g., earthquake, flood, or infrastructure collapse), the terrestrial communication infrastructure connecting GCSs or shelters may become partially or entirely inoperable. Although optimization methods can determine the GCS locations to maximize coverage and accessibility, establishing direct ground-based communication links (e.g., fiber, cellular BSs, or microwave backhaul) is often infeasible due to physical damage and environmental constraints. To restore coordination and situational awareness, a set of drones can be deployed to form a resilient airborne relay network that enables reliable inter-GCS communication.

Let $G=\left\{G_{1},G_{2},\ldots ,G_{n}\right\},\;\mathrm{and}\;U=\left\{U_{1},U_{2},\ldots ,U_{N}\right\}$ denote the sets of GCSs and drones, respectively. Each drone $U_{i}$ authenticates with its nearest GCS $G_{j}$, forming an *Authenticated_Drone_List (ADL)* given by: ${\mathrm{ADL}}_{j}=\left\{U_{i}\in U|\mathrm{Auth}\left(U_{i},G_{j}\right)=1\right\}.$

Only authenticated drones are permitted to participate in network operations. The authenticated drones are subsequently organized into clusters using a metaheuristic-based optimization process such that: $C_{j}=\left\{C_{j1},C_{j2},\ldots ,C_{jm_{j}}\right\},$ where each cluster $C_{jk}$ is managed by a designated *CH*. To ensure integrity, authenticity, and trustworthiness, the GCS issues a digitally signed *Cluster_Authentication_Descriptor (CAD)* to each selected CH.

The *intra-GCS optimization objective* is to minimize the communication cost while ensuring energy efficiency, trustworthiness, and connectivity, which is given by:

(8) $$\min_{C_{j}}\left(E_{\mathrm{intra}}+D_{\mathrm{intra}}\right),$$

subject to the following constraints:

1. *Authentication constraint: *$Auth(U_{i})=1$, for all drones $U_{i}$.
2. *Trust constraint: *$T\left(U_{i}\right)\geq T_{\mathrm{thr}}$, for all drones $U_{i}$.
3. *Unique cluster association: *$\sum_{k}Con_{ik}=1$, for each drone $U_{i}$.

Here, $E_{\mathrm{intra}}$ denotes the total intra-cluster communication energy, including the transmission and reception costs between CMs and their CHs, whereas $D_{\mathrm{intra}}$ denotes the average intra-cluster communication delay.

Minimizing this combined cost ensures energy-efficient and low-latency communication within each GCS domain. Once stable clusters are formed, the selected CHs serve as *relay nodes* to restore inter-GCS connectivity. The inter-GCS relay network is modeled as a graph H=(V,E), where $V=G\cup C_{H}$ and E denotes the set of secure links between CHs. The *global objective* is to minimize the total communication delay and energy consumption across the network:

(9) $$\min_{E}\left(D_{\mathrm{inter}}+\lambda E_{\mathrm{inter}}\right),$$

subject to the following constraints:

1. ***Inter-GCS connectivity constraint:*** $Con(G_{i},G_{j})=1$, for all required GCS pairs $\left(G_{i},G_{j}\right)$.
2. *Authentication constraint:* only authenticated CHs may participate in relay formation, i.e., $Auth\left(CH_{p}\right)=Auth\left(CH_{q}\right)=1$.

Here, Con(·) denotes the connectivity status, while Auth(·) denotes the authentication status. Both are binary indicators that take the value 1 when the corresponding condition is satisfied and 0 otherwise. Furthermore, $E_{\mathrm{inter}}$ and $D_{\mathrm{inter}}$ represent the total energy consumption and end-to-end delay of multi-hop CH–CH relay communication between GCSs, respectively. The weighting factor λ balances the trade-off between latency and energy efficiency in the overall optimization.

Overall, the proposed problem is formulated as a two-tier optimization task:

1. Intra-GCS clustering: secure, trust-aware Cluster Formation minimizing local delay and energy.
2. Inter-GCS relay formation: energy-efficient multi-hop communication among GCSs via authenticated CHs.

## 5. Proposed WAY-CR Framework

The WAY-CR framework is designed to provide secure, energy-efficient, and resilient drone-assisted communication in post-disaster environments where conventional ground-based networks become inoperable or unavailable.

**Figure 2:**
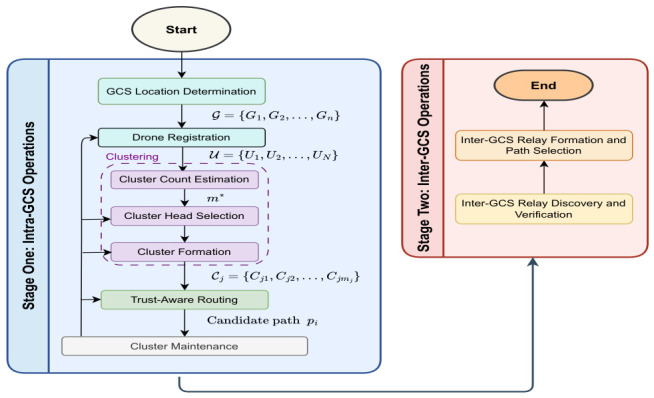
The two-stage operational workflow of the proposed WAY-CR framework.

The framework adopts a two-stage approach. In the first stage, drones undergo secure registration with their nearest GCS through an authentication process and are organized into clusters using the proposed metaheuristic-optimizer algorithm. Each CH is responsible for managing intra-cluster communication, data aggregation, and Trust-Aware Routing. An adaptive maintenance mechanism continuously monitors cluster stability, accounting for drone mobility, energy difference, and dynamic trust levels. In the second stage, the framework establishes inter-GCS communication by designating selected CHs as relay nodes. These CHs form authenticated CH-to-CH relay links, creating multi-hop airborne paths (Inter-GCS relay links) that restore communication among GCSs. This ensures end-to-end data delivery and enables mission coordination to resume across isolated GCSs. The overall workflow of WAY-CR, shown in Figure 2, highlights the two-stage structure and the interaction between Cluster Formation, Trust-Aware Routing, and inter-GCS relay establishment.

Following the background description of the WAA metaheuristic optimizer, we discuss the proposed WAY algorithm, which enhances the original WAA by replacing its stochastic phase-switching mechanism with a smooth, deterministic Yo-Yo motion controller. Then, the two main stages of the WAY-CR framework are presented in detail.

### 5.1. Background: WAA Meta-Heuristic Optimizer

**Figure 3:**
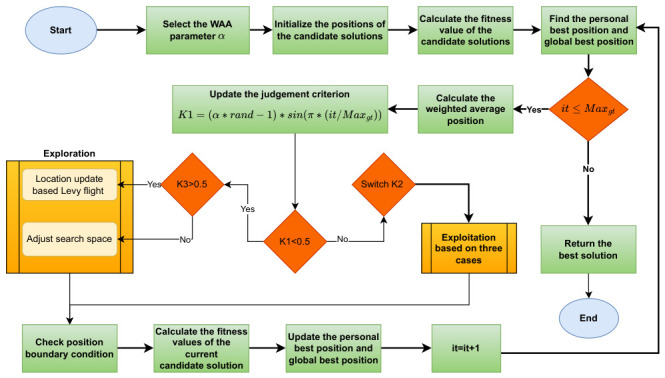
Flow diagram of the Weighted Average Algorithm (WAA).

The Weighted Average Algorithm (WAA), introduced by Cheng and De Waele [71], is a population-based meta-heuristic designed for complex nonlinear and high-dimensional optimization problems. Since it is gradient-free, WAA is suitable for objective functions that are non-differentiable or prone to local-optimum trapping. The overall workflow of WAA is illustrated in Figure 3.

In its original form, WAA uses three random selectors, $k_{1}$, $k_{2}$, and $k_{3}$, to switch between exploration and exploitation strategies. Although this mechanism helps maintain population diversity, it also introduces abrupt and fully stochastic behavioral transitions. In constrained FANET environments, such abrupt switching may be undesirable because clustering decisions must remain consistent with energy, trust, and coverage requirements.

To address this limitation, we developed an enhanced WAA variant called WAY. The proposed optimizer replaces the original stochastic switching mechanism with a unified Yo-Yo motion-based search operator, as described next.

### 5.2. Proposed WAY Optimizer Algorithm

The proposed WAY optimizer (Yo-Yo Motion-Embedded WAA) is a novel enhanced variant of the WAA optimizer, which embeds the Yo-Yo motion concept into WAA for enhanced performance. The Yo-Yo Motion-Based Operator represents the fundamental movement mechanism of the proposed WAY optimizer. This operator is inspired by the physical *Yo-Yo* motion phenomenon, characterized by a cyclic pattern of expansion and contraction [72]. In meta-heuristic optimization, this behavior is modeled as an oscillatory movement of agents around a guidance point, allowing the algorithm to alternate between exploration and exploitation phases during the search process. The WAY replaces the three stochastic selectors $\left(k_{1},\;k_{2},\;k_{3}\right)$ used in WAA to alternate between exploration strategies (Lévy jumps or random restarts) and three exploitation cases with a Yo-Yo Motion-Based Operator, which models the search dynamics as a continuous, oscillatory process that governs search dynamics over time. This unified mechanism eliminates the need for hard phase switching, enhances stability, and preserves the algorithm’s global search capability. The primary objective of WAY is to improve the stability and convergence behavior of the algorithm by introducing a continuous, oscillatory movement model inspired by Yo-Yo dynamics. While the WAA alternates between discrete exploration and exploitation phases, WAY replaces this binary switching mechanism with a unified motion operator that naturally oscillates between both behaviors. This design makes WAY especially suitable for constrained and dynamic FANET scenarios.

#### Algorithm Description

Given an objective f(x), population size *N*, dimension *d*, and a maximum of $T_{\max}$ iterations, the algorithm begins by randomly initializing candidate solutions $X_{i}^{t=1}\in R^{d}$ for i=1,…,N. It then evaluates their fitness $f(X_{i}^{1})$, set personal bests $P_{i}^{1}\;=\;X_{i}^{1}$ with $f\left(P_{i}^{1}\right)\;=\;f\left(X_{i}^{1}\right)$, and the global best $G_{1}\;=\;\arg\min_{i}f\left(X_{i}^{1}\right)$. For each iteration $t=1,2,\ldots ,T_{\max}$, the current costs $c_{i}^{t}=f\left(X_{i}^{t}\right)$ are evaluated and a fixed elite subset of size *n* (the *n* lowest-cost individuals) is selected.

The weighted mean (guidance center $\mu_{t}$) is computed as follows:

(10) $$\mu_{t}\;=\;\sum_{i=1}^{n}X_{i}^{t}\cdot \frac{S_{t}-c_{i}^{t}}{S_{t}\;\left(n-1\right)},\;\mathrm{where}\;S_{t}\;=\;\sum_{i=1}^{n}c_{i}^{t}$$

The weights sum to one by construction since $\sum_{i=1}^{n}\left(S_{t}-c_{i}^{t}\right)=S_{t}\left(n-1\right)$. After generating new candidates from $\mu_{t}$ (per the chosen update rule), we update personal bests $P_{i}^{t+1}$ when improved and the global best $G_{t+1}$. The final output is given by $X_{\mathrm{best}}=G_{T_{\max}}$.

**Damped Yo-Yo control signal:** A key feature in WAY is the damped Yo-Yo controller, which replaces WAA’s random phase selectors with a structured oscillatory signal:

(11) $$k_{1}\left(t\right)=C+A\;e^{-\beta_{\mathrm{damp}}\tau }\;\sin\left(\omega \tau +\phi \right),\;\tau =t/T_{max}\in \left(0,1\right].$$

where *C* is a constant offset that defines the baseline level of the control signal, *A* and ω define the burst amplitude and frequency, while $\beta_{\mathrm{damp}}$ reduces oscillations over time, naturally biasing the run toward exploitation as *t* increases. The search phase is determined by the value of $k_{1}\left(t\right)$ as follows: $k_{1}\left(t\right)<0.5\;\Rightarrow \;\mathrm{Exploration},\;k_{1}\left(t\right)\geq 0.5\;\Rightarrow \;\mathrm{Exploitation}.$

Unlike WAA’s random selector $k_{1}$, this rule produces a structured and gradually damped oscillation, ensuring a smooth transition from global exploration in early iterations to focused exploitation in later iterations. As *t* increases, $\mu_{t}$ concentrates and $k_{1}\left(t\right)$ stabilizes toward exploitation, improving convergence stability and reducing abrupt search transitions seen in the original WAA.

**WAY update operator:** The WAY update rule eliminates the stochastic selectors $k_{2}$ and $k_{3}$ and unifies the exploitation mechanism into a single convex pull. The update rule is defined as follows:

(12) $$X_{i}^{t+1}=\left\{\begin{array}{cc} G_{t}+L_{i}^{t},\; & \begin{array}{cc} \; & \mathrm{with}\;\mathrm{probability}\;\frac{1}{2},\\ \; & \mathrm{if}\;k_{1}\left(t\right)<0.5\;(Lé\mathrm{vy}\;\mathrm{exploration}), \end{array}\\ U([\ell ,u]),\; & \begin{array}{cc} \; & \mathrm{with}\;\mathrm{probability}\;\frac{1}{2},\\ \; & \mathrm{if}\;k_{1}\left(t\right)<0.5\;(\mathrm{random}\;\mathrm{restart}), \end{array}\\ \eta_{t}\left[w_{1}^{t}\mu_{t}+w_{2}^{t}\left(P_{i}^{t}-X_{i}^{t}\right)+w_{3}^{t}\left(G_{t}-X_{i}^{t}\right)\right],\; & \;\;\mathrm{if}\;k_{1}\left(t\right)\geq 0.5. \end{array}\right.$$

where $\eta_{t}\in \left(0,1\right]$ is a step size, which may be fixed or slowly decreasing, and the weights $w_{1}^{t}$, $w_{2}^{t}$, and $w_{3}^{t}$ satisfy $w_{j}^{t}=\frac{r_{j}}{r_{1}+r_{2}+r_{3}},\;r_{j}∼U\left(0,1\right),\;j\in \left\{1,2,3\right\},$ thereby promoting role diversity among collective knowledge, personal memory, and global leadership. The vectors ***ℓ*** and u denote the lower and upper bounds of the search space, respectively.

The exploration phase uses heavy-tailed Lévy steps, with scale parameter σ computed using the Mantegna formula, given by $L_{i}^{t}=\frac{\xi }{{\left|\zeta \right|}^{1/\beta }},\;\xi ∼N\left(0,\sigma^{2}I\right),\;\zeta ∼N\left(0,I\right),\;1<\beta \leq 2.$

After each update, a boundary check is applied before fitness evaluation and before updating the personal and global best solutions, in order to ensure that all candidate solutions remain within the feasible search space: $X_{i}^{t+1}\leftarrow \min\;\left(\max(X_{i}^{t+1},\ell ),u\right).$

Table 2 summarizes the key differences between the WAA and the WAY. While WAA relies on random phase switching and discrete exploration strategies, WAY replaces these with a deterministic, oscillatory controller that ensures smooth transitions between exploration and exploitation. This improves stability, reduces randomness, and supports better convergence in constrained binary search spaces such as CH selection in FANETs.

### 5.3. Stage One: Intra-GCS Operations

Stage 1 covers the intra-GCS clustering, routing, and maintenance processes, which are divided into seven key steps, given by: 1. GCS Location Determination, 2. Drone Registration, 3. Cluster Count Estimation, 4. CH selection, 5. Cluster Formation, 6. Trust-Aware Routing (TAR), and 7. Cluster Maintenance.

In this stage, once the CHs are selected, the remaining non-CH drones join their respective clusters to form a complete network structure. Subsequently, data routing is performed using the TAR mechanism, followed by an Adaptive Cluster Maintenance procedure to ensure stable and reliable intra-GCS operations. The following subsections describe each step in detail.

#### 5.3.1. Step 1: GCS Location Determination

To enhance communication efficiency and resilience in disaster scenarios, a GCS location determination module is incorporated as a pre-processing stage within the WAY-CR framework. This module identifies the positions of the communication shelters, referred to in this work as GCSs, which act as communication anchors and influence the subsequent clustering and routing phases by shaping the spatial organization of drones. Disaster scenarios exhibit substantial heterogeneity in type, scale, severity, and spatial impact [73]. Because disaster conditions evolve dynamically, jointly optimizing GCS placement in real time together with clustering, trust evaluation, and routing would introduce an additional decision layer beyond the scope of the present work. Therefore, the proposed framework does not treat GCS placement as an optimization variable. Instead, the locations of the available GCSs are assumed to be identified or assigned at deployment time and are used as external inputs to the network initialization process. These GCSs may correspond to surviving fixed stations, portable emergency GCS units, or mobile command vehicles. The minimum assumption of WAY-CR is that at least one operational or deployable GCS is available to initialize Drone Registration and local Cluster Formation. When inter-GCS communication is required, the framework further assumes that two or more GCSs are present in the affected region, but direct communication between them may be disrupted due to infrastructure damage, range limitations, or terrain obstruction. Under this condition, WAY-CR restores connectivity among disconnected GCS domains through trusted drone/CH relay discovery, relay verification, and inter-GCS path formation. Once the available GCS locations are identified, the framework proceeds to the secure integration of drones into the network. Each drone must authenticate itself with the nearest GCS before participating in clustering or communication.

#### 5.3.2. Step 2: Drone Registration

Once the GCS locations are established, drones are deployed, and each drone sends an authentication request message Auth_Request(drone_id, credentials) to the nearest GCS. To ensure confidentiality during the authentication exchange, the proposed Security Model (Section 3.5) is employed. Specifically, the identity of drones and credential parameters are encrypted using the Paillier Homomorphic Cryptosystem (PHC) before transmission, enabling secure verification without exposing plaintext data. The GCS decrypts and verifies the credentials using its private key. If verification succeeds, the drone is added to the *Authenticated_Drone_List (ADL)* with the status active and receives an Auth_OK message; otherwise, it receives an Auth_FAIL response. The ADL thus serves as the authoritative record of drones that have successfully completed encrypted identity verification through the GCS, ensuring that only legitimate and trusted drones participate in clustering and routing activities. If a drone fails authentication, it is automatically assigned a trust score of zero, thereby excluding it from both CH candidacy and routing participation.

During CH selection and route formation, an identity verification step is executed as a preliminary check using encrypted authentication records. Nodes failing authentication are not allowed to participate in any framework operations. Trust is computed (Section 3.4) only if authentication succeeds. By integrating homomorphic encryption in this phase, authentication data remains confidential and tamper-resistant, while enabling the GCS to perform necessary trust computations securely. Once a GCS has obtained a set of authenticated drones, it performs *Cluster Count Estimation* as follows.

#### 5.3.3. Step 3: Cluster Count Estimation

To ensure energy-efficient and delay-aware clustering in FANETs, the number of clusters (equivalently, the number of CHs) must be estimated before CH selection. This step is critical in the proposed framework since the cluster count directly affects communication overhead, CH workload, and the feasibility of balanced bandwidth allocation for both intra- and inter-cluster communication.

Our proposed framework follows a Cluster Count Estimation strategy that employs node connectivity, drone coverage conditions, and bandwidth balance to derive the optimal number of clusters [58]. For each drone $U_{i}\in U$(i=1,2,…,N) and candidate CH $CH_{k}\in C$(k=1,2,…,m), connectivity between them is established if the Euclidean distance $D_{ik}$ satisfies $D_{ik}$ = $\min\{D_{i1},D_{i2},\ldots ,D_{im}\}$, where *m* is the desired number of clusters and *N* is the number of drones. A binary connectivity indicator is defined as:

(13) $$Con_{ik}=\left\{\begin{array}{cc} 1, & \mathrm{if}\;D_{ik}\leq R,\\ 0, & \mathrm{otherwise}. \end{array}\right.$$

Each drone is associated with its nearest CH within range *R*, ensuring full coverage. The connectivity constraints are expressed as follows:

(14) $$\sum_{k=1}^{m}Con_{ik}=1,\;\forall U_{i}\in U,$$

(15) $$\sum_{i=1}^{N}Con_{ik}=CM_{k},\;\forall CH_{k}\in C,$$

where $CM_{k}$ denotes the number of members in cluster *k*.

Given the limited bandwidth in FANETs, intra- and inter-cluster communications must be balanced to avoid CH overloading (for small *m*) and bandwidth underutilization (for large *m*). Let $B_{ia}$ denote the available intra-cluster bandwidth, $B_{ir}$ the inter-cluster bandwidth, and $CM_{k}$ the number of members under $CH_{k}$. We impose the bandwidth-balance constraint given by:

(16) $$\frac{1}{m}\sum_{k=1}^{m}\frac{B_{ia}}{\sqrt{CM_{k}}}\;\leq \;\frac{B_{ir}}{\sqrt{m}}.$$

Assuming approximately balanced clusters, $CM_{k}\approx N/m$ for all *k*, (16) yields the upper bound:

(17) $$m\;\leq \;m_{\max}\;=\;\frac{B_{ir}}{B_{ia}}\sqrt{N}.$$

In practice, we approximate the target number of clusters by this bound, $m^{*}\approx m_{\max},$ and pass $\left\lfloor m^{*}\right\rfloor$ as the desired number of CHs to the selection algorithm, providing a simple bandwidth-aware initialization for the clustering process.

#### 5.3.4. Step 4: CH Selection

To address the optimization problem defined in Section 4, we adapt the WAY to operate over a binary search space. This section describes how candidate solutions are encoded, how constraint handling is enforced, and how the algorithm updates binary representations while maintaining feasibility, as follows:

The CH selection process in the WAY-CR framework serves a dual purpose: ensuring efficient network operation and reducing the number of drones required to maintain effective communication and secure data exchange. This process is formulated as a multi-objective optimization problem. By evaluating multiple performance criteria, including energy balance, communication costs, mobility stability, trustworthiness, and load distribution, our model selects the best subset of CHs from the available drones. These CHs are responsible for data aggregation, cluster coordination, and communication with the shelter or GCS, thereby reducing the computational and communication burden on regular drones. The WAY-Based CH Selection procedure is outlined in Algorithm 1. The proposed CH selection procedure acts as a multi-criteria decision-making mechanism that not only maximizes communication efficiency and security, but also implicitly minimizes the number of drones required for resilient FANET operations. To solve this optimization problem, we first formulate the proposed fitness function and subsequently detail the solution procedure based on the proposed WAY algorithm.

**Fitness Function Design:** The longevity and stability of a FANET significantly depend on the strategic selection of CHs, which are inherently more energy-intensive due to their responsibilities in both intra- and inter-cluster communication. Additionally, factors such as drone mobility, communication distances, and trustworthiness significantly affect overall network performance. To address these challenges, we propose a multi-objective fitness function that integrates essential network characteristics to guide the best CH selection.
**Algorithm 1:** WAY-Based CH selection
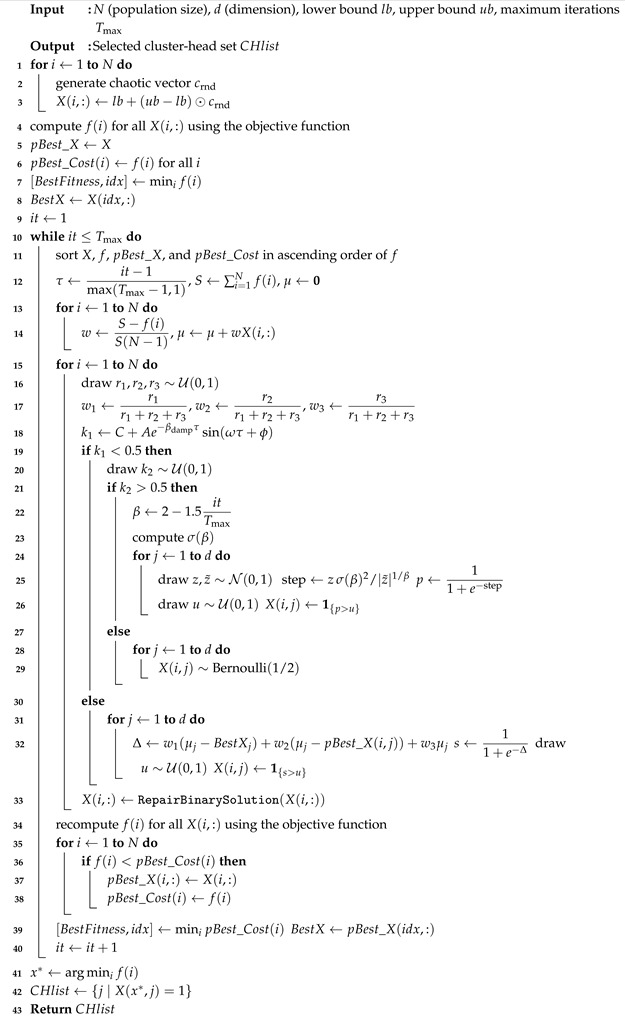


Let $CH_{k}$ denote the $k^{th}$ CH, where k∈{1,2,…,m} and *m* is the total number of clusters. Each cluster *k* contains $CM_{k}$ members, represented as $CM_{q,k}$ for the $q^{th}$ member, with $q\in \{1,2,\ldots ,CM_{k}\}$. The Euclidean distance between any two drones *i* and *j* is denoted by $Dis(U_{i},U_{j})$. The network consists of a total of *N* drones, where $n(U_{i})$ represents the number of direct neighbors (or communication links) associated with drone $U_{i}$. Let $E(U_{i})$ denote the energy of $U_{i}$, and $SV_{Ui}$ denotes the average speed variation between a drone Ui and its neighbors n(Ui). Based on these definitions, the following factors are incorporated into the fitness function:

- **Energy Consumption** f(E): For a given clustering configuration, we estimate the total communication energy spent by all drones in one reporting round, including transmission, reception, and data aggregation at the CHs. Let loss denote this total consumed energy and let $E_{\mathrm{tot}}$ be the total available network energy. The energy cost (to be minimized) is then defined as:
  (18) $$f\left(E\right)=\frac{\mathrm{loss}}{E_{\mathrm{tot}}},$$
  so that smaller values of f(E) indicate more energy-efficient clustering (more residual energy left in the network).
- **Intra-cluster Communication Distance** (f(CD)): Measures the average distance between CHs and their members to minimize communication energy consumption:
  (19) $$f\left(CD\right)=\frac{\sum_{k=1}^{m}\sum_{q=1}^{CM_{k}}Dis\left(CH_{k},CM_{q,k}\right)}{\sum_{i=1}^{N}\sum_{j=1}^{n(U_{i})}Dis\left(U_{i},U_{j}\right)}$$
- **Distance to GCS** (f(DB)): Quantifies how far the selected CHs are from the GCS by comparing their average fourth-power distance to the worst-case drone distance. A smaller value of f(DB) indicates that CHs are, on average, much closer to the GCS, which helps reduce delay and improve link reliability:
  (20) $$f\left(DB\right)=\frac{\frac{1}{m}\sum_{k=1}^{m}\mathrm{Dist}{\left(CH_{k},\mathrm{GCS}\right)}^{4}}{\max_{1\leq i\leq N}\mathrm{Dist}{\left(U_{i},\mathrm{GCS}\right)}^{4}}.$$
- **Load Balancing** f(LB): We measure load balancing with respect to two aspects: (i) the number of CMs served by each CH and (ii) the aggregate communication distance from CMs to their CHs.
  First, we quantify the uniformity of cluster sizes via Jain’s index on the CM counts ${\left\{Cn_{k}\right\}}_{k=1}^{m}$: $J_{\mathrm{count}}\left(\mathrm{Cn}\right)=\frac{{\left(\sum_{k=1}^{m}Cn_{k}\right)}^{2}}{\;m\sum_{k=1}^{m}Cn_{k}^{2}\;},\;J_{\mathrm{count}}\in \left[0,1\right].$
  This yields a count-based load-balancing cost $f_{\mathrm{LB}}^{\mathrm{count}}=1-J_{\mathrm{count}}\left(\mathrm{Cn}\right),$ so that $f_{\mathrm{LB}}^{\mathrm{count}}=0$ corresponds to perfectly balanced CM counts and larger values indicate more imbalance.
  Second, for each CH *k* we compute a distance-based load $L_{k}$ as the sum of distances from *k* to its assigned CMs, and apply Jain’s index to the load vector $L={\left\{L_{k}\right\}}_{k=1}^{m}$:
  (21) $$J_{\mathrm{dist}}\left(L\right)=\frac{{\left(\sum_{k=1}^{m}L_{k}\right)}^{2}}{\;m\sum_{k=1}^{m}L_{k}^{2}\;},\;J_{\mathrm{dist}}\in \left[0,1\right],$$
  with the corresponding distance-based cost given by $f_{\mathrm{LB}}^{\mathrm{dist}}=1-J_{\mathrm{dist}}\left(L\right).$
  The final load-balancing cost (to be minimized) is a convex combination of the two components,
  (22) $$f\left(\mathrm{LB}\right)=\gamma \;f_{\mathrm{LB}}^{\mathrm{count}}+\left(1-\gamma \right)\;f_{\mathrm{LB}}^{\mathrm{dist}},$$
  with γ=0.5 in our experiments. Hence, f(LB)∈[0,1], with 0 indicating perfectly balanced load and larger values indicating more imbalance across CHs.
- **Speed Variation (f(SV))**: Captures the relative mobility of drones in 3D space. High variation may cause unstable clustering, so low-speed deviation is preferred:
  (23) $$SV_{U_{i}}=\sqrt{{\left(sv_{U_{i}^{x}}\right)}^{2}+{\left(sv_{U_{i}^{y}}\right)}^{2}+{\left(sv_{U_{i}^{z}}\right)}^{2}}$$
  (24) $$\begin{array}{c} sv_{U_{i}^{x}}=\frac{1}{n(U_{i})}\sum_{j=1}^{n(U_{i})}\left(v_{i}\cos\theta_{i}\cos\psi_{i}-v_{j}\cos\theta_{j}\cos\psi_{j}\right)\\ sv_{U_{i}^{y}}=\frac{1}{n(U_{i})}\sum_{j=1}^{n(U_{i})}\left(v_{i}\cos\theta_{i}\sin\psi_{i}-v_{j}\cos\theta_{j}\sin\psi_{j}\right)\\ sv_{U_{i}^{z}}=\frac{1}{n(U_{i})}\sum_{j=1}^{n(U_{i})}\left(v_{i}\sin\theta_{i}-v_{j}\sin\theta_{j}\right) \end{array}$$
  (25) $$f\left(SV\right)=\frac{\sum_{k=1}^{m}SV_{CH_{k}}}{\sum_{p=1}^{N}SV_{U_{p}}}$$
- **Overall Trust (f(OT))**: Ensures that drones with a higher reputation and reliable behavior are more likely to become CHs. The trust is computed using Equation (5).

To unify these metrics into a single fitness score, each normalized objective function $f^{\prime }\left(X\right)$ is weighted by a tunable parameter $\zeta_{i}$ such that $\sum_{i=1}^{6}\zeta_{i}=1$. The overall CH fitness value is then computed as follows:

(26) $$fit_{CH}=e^{-(\zeta_{1}\left(1-f^{\prime }\left(E\right)\right)+\zeta_{2}\left(1-f^{\prime }\left(CD\right)\right)+\zeta_{3}\left(1-f^{\prime }\left(DB\right)\right)+\zeta_{4}\left(1-f^{\prime }\left(LB\right)\right)+\zeta_{5}\left(1-f^{\prime }\left(SV\right)\right)+\zeta_{6}f^{\prime }\left(OT\right))}$$

To ensure fair contribution of each objective in the fitness calculation, all fitness components are normalized to a common scale before applying the weights. The normalization is performed using min-max scaling as shown:

(27) $$f^{\prime }\left(X\right)=\frac{f\left(X\right)-f{\left(X\right)}_{\min}}{f{\left(X\right)}_{\max}-f{\left(X\right)}_{\min}}$$

where f(X) is the raw value of the objective function, $f{\left(X\right)}_{\min}$ and $f{\left(X\right)}_{\max}$ are the minimum and maximum values of that function observed in the population during evaluation, and $f^{\prime }\left(X\right)\in \left[0,1\right]$ is the normalized value used for weighted computation.

While the core fitness ${\mathrm{fit}}_{CH}$ captures the quality of a candidate CH set based on the underlying performance metrics (lower is better), we further scale it by a soft trust factor. Let ρ∈[0,1] be the *trust ratio*, defined as the fraction of selected CHs whose trust value exceeds a given threshold. The *full objective* (to be minimized) is therefore given by:

(28) $${\mathrm{fit}}_{CH}^{\mathrm{obj}}={\mathrm{fit}}_{CH}\;\left(1+\alpha (1-\rho )\right)\;,$$

where α≥0 is a tunable weight that controls how strongly the objective is penalized when the trust ratio ρ is low, with larger values of α placing more emphasis on trust.

**WAY-Based CH Selection Operations:** The proposed WAY-based optimizer performs an iterative, population-based search that balances exploration and exploitation through the Yo-Yo motion embedding into the WAA. In this work, the WAY is adapted to a *binary form* suitable for CH selection in FANETs, where a sigmoid transfer function maps continuous position updates into binary states. To enhance robustness under dynamic drone mobility, a stochastic exploration strategy is incorporated using Lévy-inspired bit flips and partial reinitialization, ensuring diversity in the binary search space. Exploration and exploitation are adaptively controlled by a sinusoidal time-varying function that alternates the search between global and local modes. Only drones that appear in the *Authenticated_Drone_List (ADL)* participate in the CH selection process. The complete computational flow of the proposed WAY-Based CH Selection is formally presented in Algorithm 1. The steps are detailed as follows:

- **Problem encoding:** Let the number of drones be *n* and the search dimension be d=n. Each candidate solution is a binary vector $x=\left[x_{1},x_{2},\ldots ,x_{d}\right],\;x_{k}\in \left\{0,1\right\},$ where $x_{k}=1$ indicates that drone $U_{k}$ is selected as a CH, and $x_{k}=0$ indicates that it is a CM. A feasible solution must contain at least one CH.
- **Initialization:** Given the drones count *N*, lower and upper bounds (lb,ub), and maximum iterations $T_{\max}$, the initial population is generated as follows:

For each individual i=1,…,N, generate a chaotic random vector $c_{\mathrm{rnd}}\in {\left[0,1\right]}^{d}$ and set $x_{i}\leftarrow lb+\left(ub-lb\right)\times c_{\mathrm{rnd}}.$ Then, apply a binarization step (e.g., $x_{i,j}\leftarrow 1$ if $x_{i,j}\geq 0.5$, else 0) to obtain $x_{i}\in {\left\{0,1\right\}}^{d}$. Evaluate all individuals using the clustering objective in (28) and denote the resulting fitness values by $f_{i}$. Initialize it←1, and set (BestX,BestFitness) to the best individual in this initial population.

- **Main optimization loop:** While $it\leq T_{\max}$, the algorithm updates the population as:

(i) **Sorting and guidance center construction:** Sort the population in ascending order of fitness, such that $f_{\left(1\right)}\leq f_{\left(2\right)}\leq \cdots \leq f_{\left(N\right)}$, with corresponding solutions $x_{\left(1\right)},\ldots ,x_{\left(N\right)}$. Compute the sum of fitness values $S=\sum_{i=1}^{N}f_{\left(i\right)}$ and the normalized iteration counter τ given by: $\tau =\frac{it-1}{\max(T_{\max}-1,1)}$ Initialize $\mu \leftarrow 0\in R^{d}$ and form the weighted guidance center:
  (29) $$\mu \leftarrow \sum_{i=1}^{N}x_{\left(i\right)}\;\frac{S-f_{\left(i\right)}}{S(N-1)}.$$
(ii) **WAY-based branching for each individual:** For each individual *i*, compute the time-varying control parameter: $k_{1}=C+A\;e^{-\beta_{\mathrm{damp}}\tau }\;\sin\left(\omega \tau +\phi \right),$ and draw $r_{1},r_{2},r_{3}∼U\left(0,1\right)$ to obtain normalized weights $w_{j}=\frac{r_{j}}{r_{1}+r_{2}+r_{3}},\;\mathrm{for}\;j=1,2,3.$ If $k_{1}<0.5$, the algorithm applies an *exploration* update to individual *i*; otherwise, it applies an *exploitation* update.
(iii) **Exploration branch ($k_{1}<0.5$):** Sample $k_{2}∼U\left(0,1\right)$ to select the exploration mode.
  - **Lévy-inspired exploration ($k_{2}>0.5$):** Define the iteration-dependent shape $\beta_{t}=2-1.5\frac{it}{T_{\max}},$ and its scale $\sigma_{\beta_{t}}=\frac{\Gamma \left(1+\beta_{t}\right)\;\sin\left(\pi \beta_{t}/2\right)}{\beta_{t}\;\Gamma \left(\frac{1+\beta_{t}}{2}\right)\;2^{(\beta_{t}-1)/2}}.$
    For each dimension j=1,…,d, $z_{j},{\tilde{z}}_{j}∼N\left(0,1\right),\;{\mathrm{step}}_{j}=z_{j}\frac{\sigma_{\beta_{t}}^{2}}{|{\tilde{z}}_{j}{|}^{1/\beta_{t}}},\;p_{j}=\frac{1}{1+e^{-{\mathrm{step}}_{j}}},$ then with $u_{j}∼U\left(0,1\right)$, update
    (30) $$x_{i,j}^{t+1}=\left\{\begin{array}{cc} 1, & p_{j}>u_{j},\\ 0, & \mathrm{otherwise}. \end{array}\right.$$
  - **Random restart ($k_{2}\leq 0.5$):** Draw a single Bernoulli random variable $b∼\mathrm{Bernoulli}\;\left(\frac{1}{2}\right)$ and set $x_{i,j}^{t+1}=b,\;j=1,\ldots ,d.$
(iv) **Exploitation branch ( $k_{1}\geq 0.5$):** In exploitation, individual *i* is guided simultaneously by the current center μ, the current global best BestX, and its personal best ${\mathrm{pBest}}_{X}\left(i,\cdot \right)$. For each dimension j=1,…,d,
  (31) $$\Delta_{i,j}=w_{1}\left(\mu_{j}-{\mathrm{BestX}}_{j}\right)+w_{2}\left(\mu_{j}-{\mathrm{pBest}}_{X}\left(i,j\right)\right)+w_{3}\mu_{j},\;$$
  and
  (32) $$x_{i,j}^{t+1}=\left\{\begin{array}{cc} 1, & \frac{1}{1+e^{-\Delta_{i,j}}}>u∼U\left(0,1\right),\\ 0, & \mathrm{otherwise}. \end{array}\right.$$
(v) **Constraint enforcement and re-evaluation:** After either branch, the updated individual is checked for feasibility as follows:
  (a) **CH repair strategy:** To avoid degenerate solutions with too few CHs, a repair operator is applied whenever a candidate solution $b_{i}\in {\left\{0,1\right\}}^{N}$ contains at most one CH. Let $C=\{u|b_{i}\left(u\right)=1\},\;m=|C|,$ and let $m^{*}$ denote the target number of CHs estimated in Step 3 (Cluster Count Estimation). If m>1, the solution is left unchanged. For N=1, the single node is trivially set as CH. For N>1, we first determine an effective upper bound on the CH count, $n_{\mathrm{CH}}^{\mathrm{eff}}=\min\left(\left\lfloor m^{*}\right\rfloor ,N\right),$ and then randomly choose a target CH count $k_{\mathrm{CH}}$ in the range $\left[2,\;n_{\mathrm{CH}}^{\mathrm{eff}}\right]$ (or $k_{\mathrm{CH}}=n_{\mathrm{CH}}^{\mathrm{eff}}$, if $n_{\mathrm{CH}}^{\mathrm{eff}}<2$).
    If m=0, we start with C=∅ and consider all nodes as candidates. If m=1, we keep the existing CH $u_{0}$, set $C=\{u_{0}\}$, and block $u_{0}$ and its neighbors $N(u_{0})$ (from the neighbor list) from being selected again. In both cases, the initial candidate set is the set of nodes that are not blocked.
    We then iteratively select new CHs from the current candidate set: at each step, a node *k* is chosen at random, added to C, and both *k* and its neighbors N(k) are removed from the candidates. This process continues until either no candidates remain or $\left|C\right|=k_{\mathrm{CH}}$. If after this process |C|<2 while N>1 and $n_{\mathrm{CH}}^{\mathrm{eff}}\geq 2$, one additional CH is randomly selected from the non-CH nodes to guarantee m>1. Finally, the repaired solution is defined by:
    (33) $$b_{i}\left(u\right)=\left\{\begin{array}{cc} 1, & u\in C,\\ 0, & \mathrm{otherwise}. \end{array}\right.$$
    This operator enforces a minimum of two CHs when possible, promotes spatial separation between CHs by blocking their neighbors, and keeps the number of CHs close to the bandwidth-aware target $m^{*}$ implied by the cluster-count estimation step (Section 5.3.3).
  (b) **Update and re-evaluation:** The updated population is then re-evaluated using Equation (28); if a solution improves on BestFitness, then (BestX,BestFitness) are replaced by this solution. Finally, the iteration counter is incremented: it←it+1.

- **Termination and output.** When $it>T_{\max}$, the algorithm selects the solution with the minimum fitness, $x^{*}=\arg\min_{x_{i}}f_{i},$ and extracts the final CH set as *CHlist* = $\left\{k|x_{k}^{*}=1\right\}$. The list *CHlist* is returned as the final CH configuration.

#### 5.3.5. Step 5: Cluster Formation

After the CHs are selected using the WAY-Based CH Selection procedure, the remaining drones, referred to as CMs, must associate with suitable CHs to form fully functional clusters. This stage transforms the optimized CH set into a structured and connected cluster topology. The process is entirely decentralized, enabling each drone to make autonomous decisions based on locally observable information and periodic broadcasts from nearby CHs.

Each GCS generates a signed *Cluster_Authentication_Descriptor (CAD)* containing the cluster ID, selected CH, potential members, CH neighbor list, timestamp, and digital signature. The CAD is encrypted using the PHC and distributed to the designated CH. Upon receiving the CAD, the CH verifies the GCS signature to confirm authenticity and broadcasts a CH_Announcement to nearby drones. Each drone independently selects its CH through the following decentralized procedure:

(i) Local Table Construction: Each drone compiles a local table of candidate CHs based on the received announcement information CH_Announcement.
(ii) Scoring and Selection: Each drone evaluates every candidate CH (*s*) using a multi-criteria score:
  (34) $$\mathrm{Score}\left(s\right)=\alpha_{1}\left(1-{\mathrm{NormDist}}_{s}\right)+\alpha_{2}\cdot {\mathrm{LinkStability}}_{s}+\alpha_{3}\cdot {\mathrm{Trust}}_{s}+\alpha_{4}\cdot \left(1-\frac{E_{\mathrm{tx}}\left(s\right)}{E^{\mathrm{residual}}}\right)$$
  where ${\mathrm{NormDist}}_{s}$ denotes the normalized Euclidean distance between the drone and the candidate CH *s*, ensuring that nearer CHs are preferred. ${\mathrm{LinkStability}}_{s}$ quantifies the reliability of the communication link. It is defined between any two drones *i* and *j* as an exponentially weighted function of their relative velocity and mutual distance:
  (35) $$LS_{ij}=\exp\;\left(-\frac{|v_{i}-v_{j}|}{V_{\max}}\right)\times \exp\;\left(-\frac{d_{ij}}{R_{c}}\right)$$
  where $v_{i}$ and $v_{j}$ denote the speeds of drones *i* and *j*, respectively, $V_{\max}$ represents the maximum drone velocity (used for normalization), $d_{ij}$ is the Euclidean distance between the two drones, and $R_{c}$ is the nominal communication range. Higher values of $LS_{ij}$ indicate stronger and more stable communication links. ${\mathrm{Trust}}_{s}$ represents the CH’s trustworthiness as determined by the proposed trust model. $E_{\mathrm{tx}}\left(s\right)$ is the estimated transmission energy required to reach CH *s*, and $E^{\mathrm{residual}}$ is the drone’s remaining energy. Their ratio promotes energy-efficient associations by favoring CHs that require lower transmission costs relative to available power. The drone then selects its CH as: $s^{*}=\arg\max_{s\in {\mathrm{CH}}_{\mathrm{reachable}}}\mathrm{Score}\left(s\right).$
(iii) Trust-Aware Registration: After selecting $s^{*}$, each drone encrypts its registration request and trust parameters using PHC before transmission, and then sends a registration request to the chosen CH.
  Upon receiving the request, the CH verifies the CM’s trust level using recent trust updates. Furthermore, the CH checks whether the drone is listed in the CAD and whether its encrypted trust scores satisfy the threshold condition $T\left(U_{i}\right)\geq T_{\mathrm{thr}}$. Only CMs that are both authenticated and have trust values exceeding a predefined trust threshold are admitted into the cluster. This mutual trust validation mechanism prevents compromised or low-trust drones from joining the cluster, ensuring that authentication and trust collectively safeguard intra-cluster communication.

Once all drones complete the selection and verification process, the network self-organizes into a set of clusters, with each CH acting as a local coordinator responsible for intra-cluster communication, data aggregation, and forwarding toward the GCS. This trust-aware clustering mechanism ensures that clusters are energy-balanced, secure, and dynamically maintained as drones move or as environmental conditions change, providing a stable and resilient foundation for subsequent routing and data delivery phases.

#### 5.3.6. Step 6: Trust-Aware Routing (TAR)

In the WAY-CR framework, the routing phase plays a critical role in establishing secure, energy-efficient, and mobility-aware communication between drones and the GCS. Following the clustering and CH selection process, this phase ensures that data generated by CMs is reliably forwarded through selected CHs to the GCS. After clustering, the routing stage in the WAY-CR framework establishes multi-hop communication between CHs and GCSs to restore connectivity among shelters. In this stage, drones act as aerial relays that must maintain stable, secure, and energy-efficient links under highly dynamic network conditions. Deterministic routing schemes can quickly become unstable when the topology changes; therefore, the mechanism should be probabilistic and adaptive.

To address this, the WAY-CR employs a Unified trust-aware Boltzmann distribution-based path selection approach. The proposed routing algorithm is outlined in Algorithm 2. Each candidate route is assigned a probabilistic weight based on its normalized fitness, balancing exploitation (selecting the best-known path) and exploration (considering alternative stable routes). This mechanism mitigates premature convergence to suboptimal paths and enhances resilience under frequent link fluctuations. Before path fitness evaluation, each candidate route undergoes a CAD-based neighbor consistency check. For every CH pair $\left(CH_{a},CH_{b}\right)$ along the path, both nodes must appear in each other’s *Neighbor_List* field. Only topology-consistent and mutually authenticated CH pairs are considered for trust-aware path selection. This ensures that routing paths are constructed exclusively over verified CH-CH links, preventing falsified or mismatched connections. Each candidate path $p_{i}$ is then evaluated using three key normalized metrics: $f^{\prime }\left(CT\right)$ denotes the *normalized average trust* along the path, reflecting its reliability and resistance to malicious or failing nodes; $f^{\prime }\left(E\right)$ represents the *normalized average residual energy* of drones along the path, ensuring balanced power consumption and prolonged network lifetime; and $f^{\prime }\left(LS\right)$ measures the *normalized link stability*, estimating the expected duration of link availability based on drone mobility and signal strength dynamics. The composite path fitness is defined as:

(36) $$f_{\mathrm{path}}=\left(\lambda_{1}f^{\prime }\left(CT\right)+\lambda_{2}f^{\prime }\left(E\right)+\lambda_{3}f^{\prime }\left(LS\right)\right)\times {\left[f^{\prime }\left(CT\right)\right]}^{\beta },$$

where $\lambda_{1}+\lambda_{2}+\lambda_{3}=1$ are the weighting coefficients, and β is the trust amplification parameter. The multiplicative term ${\left[f^{\prime }\left(CT\right)\right]}^{\beta }$ strengthens the influence of trust: when β>1, unreliable paths are heavily penalized; when β<1, trust sensitivity is softened to tolerate minor estimation noise.

Each path’s selection probability follows the Boltzmann distribution:

(37) $$P\left(p_{i}\right)=\frac{e^{f_{{\mathrm{path}}_{i}}/T}}{\sum_{k=1}^{N_{p}}e^{f_{{\mathrm{path}}_{k}}/T}},$$

where *T* is the temperature coefficient controlling randomness. A high *T* encourages exploration by allowing more candidate paths to be selected, while a low *T* favors exploitation of high-fitness, stable, and trusted routes.

A two-level tie-breaking rule is applied to ensure efficient routing decisions:

(i) If two or more candidate paths have similar fitness values, i.e., $|f_{{\mathrm{path}}_{i}}-f_{{\mathrm{path}}_{j}}|<\varepsilon$, then the path with the fewer hop count is preferred to reduce latency.
(ii) If such paths also have the same hop count, one of them is selected randomly to maintain path diversity and load balance.

Algorithm 2 provides a balanced tradeoff between security, endurance, and robustness, the key requirements for drone-based communication restoration between GCSs. The Boltzmann selection mechanism ensures adaptive path selection and resilience, while the two-level tie-breaking rule guarantees both efficiency and fairness in routing. While trust metrics are designed to mitigate insider threats, they operate under the assumption that all participating drones are legitimate. To uphold this assumption, authentication is enforced during the early stages of the framework, specifically in Step 2 (Section 5.3.2), before clustering and routing take place. Only authenticated drones are permitted to form or join clusters, ensuring identity validation prior to trust computation. This pre-authentication mechanism effectively blocks spoofed or unauthorized nodes from entering the network, thereby protecting the integrity of trust evaluation and ensuring that routing decisions are based solely on verified, trustworthy participants.
**Algorithm 2:** Unified Trust-Aware Boltzmann Path Selection
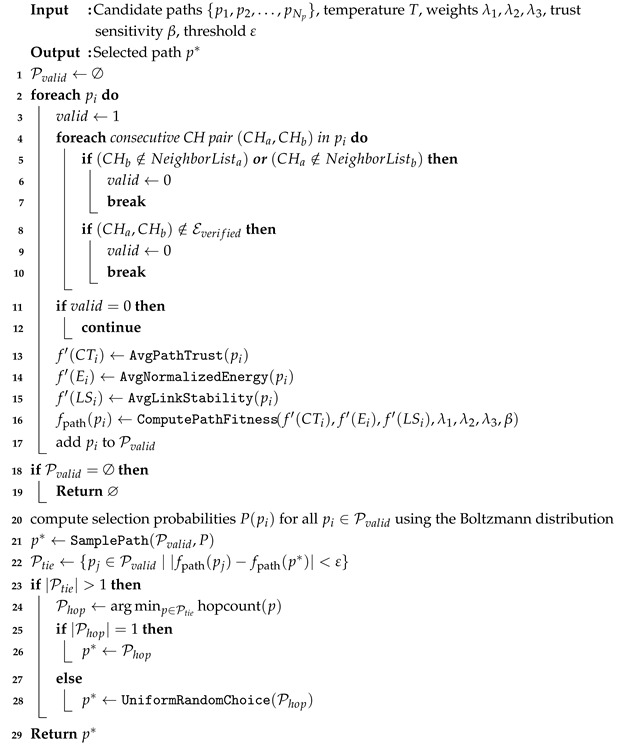

**Boltzmann Temperature Parameter Tuning for TAR:** To further enhance the adaptability of the trust-aware Boltzmann routing mechanism, the temperature parameter can be tuned online using an Upper Confidence Bound (UCB)-based Multi-Armed Bandit (MAB) strategy [74]. Instead of selecting a fixed temperature *a priori*, the controller progressively learns which temperature values provide the most effective exploration–exploitation balance under dynamic FANET conditions.

Let the discrete candidate set of temperature values be $T=\left\{T^{\left(1\right)},T^{\left(2\right)},\ldots ,T^{\left(K\right)}\right\},$ where each $T^{\left(j\right)}$ corresponds to one bandit arm. At routing epoch *t*, the controller selects one temperature arm $T_{t}\in T$ and applies it to the Boltzmann path-selection rule:

(38) $$P\left(p_{i}\right)=\frac{\exp\;\left(f_{\mathrm{path},i}/T_{t}\right)}{\sum_{k=1}^{N_{p}}\exp\;\left(f_{\mathrm{path},k}/T_{t}\right)},$$

where $f_{\mathrm{path},i}$ is the trust-aware fitness of candidate path $p_{i}$ defined by Equation (36).

Each arm is associated with an empirical mean reward ${\hat{\mu }}_{j}$ and a selection count $n_{j}$. To guarantee valid initialization, each temperature arm is selected once before the standard UCB rule is applied. After initialization, the arm selected at routing epoch *t* is determined by $a_{t}=\arg\max_{j\in \{1,\ldots ,K\}}\left({\hat{\mu }}_{j}+c\sqrt{\frac{2\ln t}{n_{j}}}\right),$ where c>0 is the exploration coefficient and $a_{t}$ is the selected arm index. The corresponding temperature used in the routing epoch is $T_{t}=T^{\left(a_{t}\right)}.$ After applying $T_{t}$, the routing controller computes the Boltzmann probabilities of feasible routing alternatives and selects the corresponding CH-to-BS routing paths. Since one routing epoch may produce one selected path for each source CH, let $S_{t}$ denote the set of successful selected paths at epoch *t*.

The UCB reward is then defined as the mean trust-aware fitness of the selected successful paths: $r_{t}=\frac{1}{|S_{t}|}\sum_{p\in S_{t}}f_{\mathrm{path}}\left(p\right),$ where $f_{\mathrm{path}}\left(p\right)$ is computed using Equation (36). If no successful routing path is obtained during epoch *t*, the reward is set to zero. Since the path fitness is constructed from normalized trust, residual energy, and link-stability terms, the resulting reward remains bounded and fully consistent with the Trust-Aware Routing objective. After observing reward $r_{t}$, the count of the selected arm is first incremented: $n_{a_{t}}\leftarrow n_{a_{t}}+1,$ and its empirical mean reward is updated as ${\hat{\mu }}_{a_{t}}\leftarrow {\hat{\mu }}_{a_{t}}+\frac{1}{n_{a_{t}}}\left(r_{t}-{\hat{\mu }}_{a_{t}}\right).$

Through this mechanism, the controller gradually identifies temperature values that yield better long-term routing behavior. In particular, the UCB policy balances exploration of underutilized temperature values with exploitation of temperatures that have previously produced high-quality routing decisions. As a result, the routing process can adapt more effectively to mobility changes, trust variation, link disruption, and fluctuating communication conditions. Temperature tuning is performed globally, meaning that one temperature value is selected per routing epoch and is applied uniformly to all routing decisions performed during that epoch. This design preserves the probabilistic structure of the Boltzmann routing policy while introducing only limited computational overhead compared with more complex contextual or per-path adaptation strategies.

#### 5.3.7. Step 7: Cluster Maintenance

To ensure robustness, energy efficiency, and trust-aware adaptation in highly dynamic FANET environments, the WAY-CR framework integrates an *Adaptive Cluster Maintenance* mechanism, as detailed in Algorithm 3. This process maintains cluster stability over time by dynamically adjusting beacon intervals, monitoring node fitness, and managing membership changes such as CH inactivity, CM disconnection, or the integration of new drones.

Each drone periodically broadcasts a beacon according to an adaptive interval $T_{b}\left(i\right)$ defined as:

(39) $$T_{b}\left(i\right)=T_{\min}+\left(T_{\max}-T_{\min}\right)\frac{e^{-\kappa M_{i}}\left(1+\beta (1-\eta_{i})\right)}{1+\beta },$$

where $M_{i}$ denotes the speed-variation metric of drone *i*, $\eta_{i}=E_{i}/E_{\max}$ is the normalized residual-energy ratio, $T_{\min}$ and $T_{\max}$ represent the minimum and maximum allowable beacon intervals, and κ,β>0 are sensitivity parameters controlling the effects of mobility and energy, respectively. This formulation is designed to decrease the beacon interval as drone mobility increases, enabling faster topology updates, and to increase the beacon interval as residual energy decreases, thereby reducing control overhead for energy-constrained nodes. Moreover, the normalization by 1+β ensures that $T_{b}\left(i\right)$ remains bounded within $\left[T_{\min},T_{\max}\right]$, providing a practical balance between network responsiveness and signaling efficiency.
**Algorithm 3:** Adaptive Cluster Maintenance
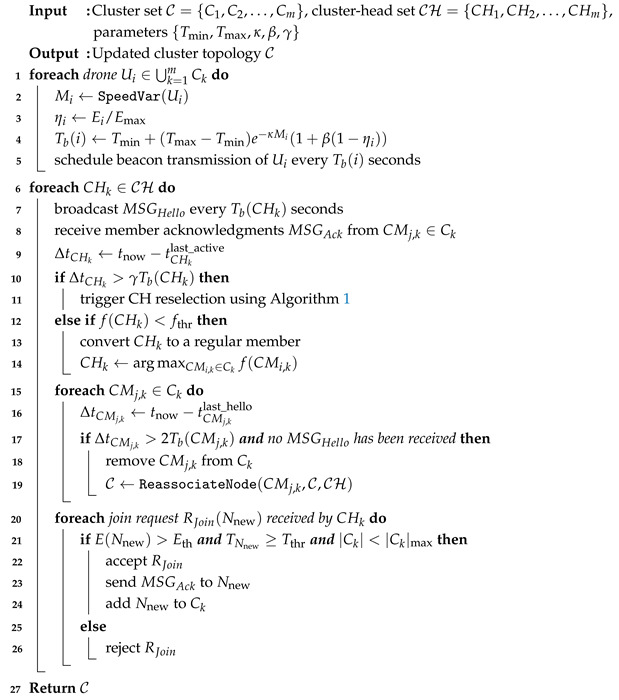


Each CH periodically transmits a MSG_Hello message to its associated CMs at the adaptive interval $T_{b}\left(CH_{k}\right)$ and updates its timestamp $t_{CH_{k}}$. CMs reply with acknowledgment messages (MSG_Ack) to confirm connectivity. If a CH exhibits inactivity, detected when the elapsed time $\Delta t_{CH_{k}}>\gamma \times T_{b}\left(CH_{k}\right)$, the node is considered inactive, and the re-selection procedure defined in Algorithm 1 is triggered. Alternatively, if the CH’s fitness value $f(CH_{k})$, as defined in Equation (26), falls below the threshold $f_{\mathrm{thr}}$, the node is demoted to a regular CM, and the member with the highest fitness is promoted as the new CH:

(40) $$CH_{k}=\arg\max_{CM_{i,k}}f\left(CM_{i,k}\right).$$

Similarly, each CM monitors its CH’s activity. If a CM fails to receive any MSG_Hello messages for more than $2\times T_{b}\left(CM_{j,k}\right)$, it is considered disconnected and triggers CM registration (as mentioned in Section 5.3.5). This enables decentralized, self-healing cluster reconfiguration without centralized coordination.

When a new or previously disconnected drone seeks to join a cluster, it sends a join request (R_Join) to the nearest available CH. The CH consults the latest ADL record to verify its authentication status. Only drones listed as active proceed to the trust and energy evaluation phase; otherwise, the CH requests re-authentication from the corresponding GCS. If the drone is present in the latest ADL, the CH then verifies its residual energy, trust value, and the current cluster capacity. A drone $N_{\mathrm{new}}$ is admitted to cluster $C_{k}$ only if its residual energy exceeds the threshold $E_{\mathrm{th}}$, its trust value is not less than the threshold $T_{\mathrm{thr}}$, and the current cluster size remains below the maximum allowed size $|C_{k}{|}_{\max}$. Otherwise, the admission request is rejected or deferred.

Finally, fitness scores for both CHs and CMs are periodically updated based on current observations of trust, residual energy, and mobility within the WAA-based multi-objective evaluation framework. These proactive updates allow the system to respond promptly to node failures, mobility-induced link disruptions, or declining trust levels, maintaining a resilient, secure, and energy-balanced cluster topology throughout the network lifecycle.

The *Authenticated_Drone_List (ADL)* serves as the authoritative record of drones that have successfully completed identity verification through the GCS. It ensures that only legitimate and trusted drones participate in clustering and routing activities throughout the WAY-CR framework. The ADL is dynamically maintained and updated as follows: During the registration phase (Step 2, Section 5.3.2), each drone submits an Auth_Request containing its credentials to the nearest GCS. Upon successful verification, the drone is added to the ADL with the status active, along with its trust value, residual energy, and the timestamp of the last authentication. The GCS periodically revalidates ADL entries. Drones that fail authentication or show degraded trust behavior are flagged as revoked and temporarily excluded from routing and clustering operations. If a drone becomes inactive, unresponsive, or malicious, its ADL status is immediately updated to revoked. Such drones must reauthenticate through the GCS before being allowed to rejoin any cluster or participate in routing.

### 5.4. Stage Two: Inter-GCS Operations

After completing ***Stage One***, all drones are organized into authenticated clusters, each governed by a CH and secured through CADs and the ADL. This establishes a trusted intra-GCS communication backbone where CMs exchange data securely under their respective GCSs. However, once network fragmentation or GCS disconnection occurs, intra-cluster communication alone becomes insufficient to maintain global coordination.

To restore full mission-level connectivity across distributed GCS domains, ***Stage Two*** introduces a secure inter-GCS relay mechanism that enables communication between geographically separated control segments through authenticated CH-CM relay links, using two integrated mechanisms: (i) the *Secure Inter-GCS Relay Discovery and Verification Algorithm* (Algorithm 4), which establishes authenticated and trusted relay links between clusters managed by different GCSs, (ii) the *Unified Trust-Aware Boltzmann Path Selection Algorithm* (Algorithm 2), which determines the most reliable and energy-efficient route across the verified relay graph. This ensures a resilient, trust-aware, and adaptive recovery process during inter-GCS reconfiguration. Inter-GCS connectivity is re-established through hierarchical multi-hop relays composed of CHs and CMs. When ${\mathrm{GCS}}_{i}$ intends to communicate with ${\mathrm{GCS}}_{j}$, the relay structure depends on spatial separation and the available network topology. Three operational scenarios are considered:

(i) **Direct CH–CH Relay:** If $CH_{i}$ and $CH_{j}$ are within mutual communication range, a single-hop relay path is established: $GCS_{i}\to CH_{i}↔CH_{j}\to GCS_{j}.$ This configuration achieves the lowest latency and minimal energy cost.
(ii) **Single-Hop Intra-Cluster Assistance:** When direct CH–CH connectivity is unavailable but an intermediate CM can bridge the link, the assisted path is formed as: $GCS_{i}\to CH_{i}\to CM_{i}↔CH_{j}\to GCS_{j}.$ This arrangement provides moderate latency with stable connectivity.
(iii) **Multi-Hop Relay via Intermediate CMs:** In sparse or partitioned topologies, multi-hop relaying extends communication over longer distances: $GCS_{i}\to CH_{i}\to CM_{i}↔CM_{j}\to CH_{j}\to GCS_{j}.$ While more energy intensive, this approach ensures robust end-to-end connectivity.

**Algorithm 4:** Secure Inter-GCS Relay Discovery and Verification
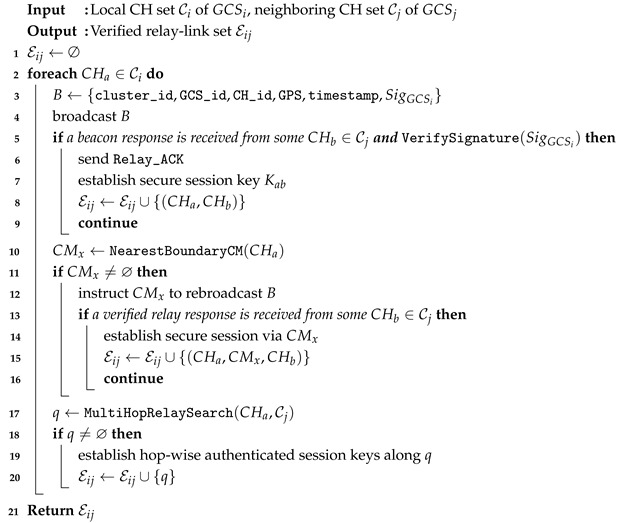


The secure establishment of these relay scenarios relies on mutual authentication between CHs belonging to different GCSs. The following subsections discuss the discovery, verification, and key exchange processes that governs this inter-GCS authentication layer in detail, as follows:

#### 5.4.1. Inter-GCS Relay Discovery and Verification

The Secure Inter-GCS Relay Discovery and Verification procedure (Algorithm 4) governs the establishment of authenticated and trusted inter-GCS relay links. CHs belonging to distinct GCSs authenticate one another using GCS-signed credentials and then form the verified communication links. Each CH holds a credential containing its identifier, trust level, and timestamp, digitally signed using the GCS’s private key. Neighboring CHs verify this credential using the public key of the issuing GCS. Only after successful mutual authentication and trust validation are the relay links added to the verified edge set $E_{verified}$ for subsequent routing.

**Scenario 1: Direct CH–CH Discovery:** Each cluster head $CH_{i}$ periodically broadcasts a signed Cluster_Beacon message:

(41) $$\mathrm{Cluster}\_\mathrm{Beacon}=\{cluster\_id,\;GCS\_id,\;CH\_id,\;GPS,\;timestamp,\;Sig_{GCS_{i}}\},$$

thereby advertising its availability for inter-GCS communication. When a neighboring $CH_{j}\in C_{j}$ receives this beacon, it verifies $Sig_{GCS_{i}}$ using the public key of ${\mathrm{GCS}}_{i}$. Upon successful verification, $CH_{j}$ replies with a Relay_ACK, acknowledging authenticity and readiness to establish a link. The mutual trust coefficient is then computed as: $CT_{ij}=\min\left(Trust\left(CH_{i}\right),\;Trust\left(CH_{j}\right)\right),$ ensuring effective trust estimation. The verified pair $\left(CH_{i},CH_{j},K_{ij}\right)$ is recorded in $E_{verified}$, providing a low-delay, energy-efficient relay channel.

**Scenario 2: Assisted CH–CM–CH Discovery:** If $CH_{i}$ and $CH_{j}$ are not within direct range, $CH_{i}$ delegates a nearby trusted cluster member ($CM_{x}$) to rebroadcast its Cluster_Beacon. The intermediate $CM_{x}$ forwards the signed beacon to $CH_{j}$, which again verifies $Sig_{GCS_{i}}$ using ${\mathrm{GCS}}_{i}$’s public key. Following validation, $CH_{j}$ returns a signed Relay_ACK, which is relayed back via $CM_{x}$. Both CHs then establish a session key $K_{ij}$ through hop-wise authenticated exchange. The verified relay tuple $\left(CH_{i},CM_{x},CH_{j},K_{ij}\right)$ is added to $E_{verified}$, providing reliable inter-GCS connectivity through adjacent clusters.

**Scenario 3: Multi-Hop CM Relay Discovery:** In sparse or fragmented environments where direct or single-hop discovery fails, controlled multi-hop propagation is used. $CH_{i}$ initiates beacon forwarding through a sequence of trusted CMs $\left(CM_{x},CM_{y},\ldots \right)$ until a remote $CH_{j}$ is reached. At each hop, integrity and trust are verified using CAD-based credentials and intra-cluster *Neighbor Lists*, preventing route falsification. Once $CH_{j}$ receives a valid beacon, it performs the same authentication and key exchange steps as Scenario 1. The resulting relay chain $\left(CH_{i},CM_{x},CM_{y},\ldots ,CH_{j}\right)$ is recorded in $E_{verified}$, providing robust communication in partitioned topologies.

**Unified Trust and Authentication Model:** Across all scenarios, the mutual trust between connected CHs is conservatively evaluated as the minimum trust value of both endpoints. Each verified link in $E_{verified}$ is secured using its session key $K_{ij}$ and authenticated through GCS-issued credentials. Only these verified, trusted links are subsequently employed within the Unified Trust-Aware Boltzmann Path Selection Algorithm (Algorithm 2) to construct secure and energy-aware inter-GCS relay routes. This process is further constrained by the ADL list and CADs, ensuring that only legitimate CHs and CMs participate in inter-GCS routing.

The procedural description of the Secure Inter-GCS Relay Discovery and Verification Process is given in Algorithm 4, which adaptively executes direct, assisted, and multi-hop relay formation based on network conditions.

#### 5.4.2. Inter-GCS Relay Formation and Path Selection

Once verified, the relay links have been established using Algorithm 4, the best inter-GCS path between ${\mathrm{GCS}}_{i}$ and ${\mathrm{GCS}}_{j}$ is determined using the Unified Trust-Aware Boltzmann Path Selection Algorithm (Algorithm 2). Each candidate path $p_{i}$ is evaluated using three normalized metrics: $f^{\prime }\left(CT_{i}\right)$—average trust of CHs along the path, $f^{\prime }\left(E_{i}\right)$—normalized residual energy, and $f^{\prime }\left(LS_{i}\right)$—average link stability.

Before path fitness evaluation, each consecutive CH pair $\left(CH_{a},CH_{b}\right)$ is validated:

- For intra-GCS links, CAD-based *Neighbor List* cross-checking ensures mutual inclusion.
- For inter-GCS links, authenticity is confirmed via verified Cluster_Beacon/ RELAY_ACK pairs in $E_{verified}$.

Each valid path’s fitness $f_{{\mathrm{path}}_{p_{i}}}$ is calculated using Equation (36), and its selection probability $P(p_{i})$ is derived using Equation (37). This Boltzmann-based probabilistic mechanism ensures that higher-fitness paths are favored while maintaining exploration to avoid route overutilization, thereby enhancing reliability and load distribution during inter-GCS recovery. By integrating secure relay discovery with probabilistic TAR, ***Stage Two*** ensures that inter-GCS communication remains both adaptive and resilient, even under dynamic topological or environmental disruptions.

### 5.5. Computational Complexity Analysis

The computational complexity of the proposed WAY-CR framework can be analyzed by decomposing it into its major operational components, namely: secure registration and clustering, Trust-Aware Routing, adaptive maintenance, and inter-GCS relay discovery. Let *n* denote the total number of drones, *m* the number of selected CHs, *N* the optimizer population size, *d* the search-space dimension, $T_{\max}$ the maximum number of optimization iterations, $N_{p}$ the number of candidate routing paths, and *L* the average hop length of a candidate path.

In the first stage, the dominant cost arises from the WAY-Based CH Selection algorithm. During each iteration, the population is sorted in O(NlogN) time, the weighted mean and position updates are computed in O(Nd) time, and the fitness values are re-evaluated in $O(NC_{f})$ time, where $C_{f}$ is the cost of evaluating the objective function for one candidate solution. Therefore, the total complexity of the CH selection process is

(42) $$C_{\mathrm{CH}}=O\;\left(T_{\max}\left(N\log N+Nd+NC_{f}\right)\right).$$

If the objective evaluation is linear in the dimension, i.e., $C_{f}=O\left(d\right)$, then this reduces to

(43) $$C_{\mathrm{CH}}=O\left(T_{\max}N\left(d+\log N\right)\right)\approx O\left(T_{\max}Nd\right).$$

After CH selection, Cluster Formation requires associating drones with selected CHs. Under a straightforward implementation where each drone checks all CHs, the complexity is $C_{\mathrm{CF}}=O\left(nm\right).$ Secure Drone Registration with the nearest GCS is performed once per drone and contributes a linear cost of $C_{\mathrm{REG}}=O\left(n\right).$ For routing, the Unified Trust-Aware Boltzmann Path Selection algorithm evaluates each candidate path by verifying consecutive CH pairs and computing trust, residual energy, and link stability. If each path contains on average *L* hops, then the complexity is $C_{\mathrm{TAR}}=O\left(N_{p}L\right),$ since path validation and metric aggregation are both linear in the path length, and the Boltzmann probability computation adds only $O(N_{p})$ overhead.

The Adaptive Cluster Maintenance procedure scans drone states, updates beacon intervals, checks CH activity, and handles re-association events. For one maintenance cycle, the complexity is linear in the number of drones and CHs: $C_{\mathrm{MAINT}}=O\left(n+m\right)\approx O\left(n\right).$ If re-election of CHs is triggered *R* times during operation, the maintenance overhead becomes $C_{\mathrm{MAINT}-\mathrm{total}}=O\left(Hn+R\;C_{\mathrm{CH}}\right),$ where *H* is the number of maintenance rounds.

In the second stage, the secure inter-GCS relay discovery and verification algorithm operates over neighboring CHs belonging to adjacent GCS regions. Let $m_{i}$ and $m_{j}$ denote the number of CHs in two neighboring GCS domains. Direct CH-to-CH discovery requires, in the worst case, checking possible relay opportunities across CH pairs, giving $C_{\mathrm{DIR}}=O\left(m_{i}m_{j}\right).$ If assisted or multi-hop relay discovery is required through CMs, the search behaves like a graph exploration over boundary relay nodes. Let $V_{b}$ and $E_{b}$ denote the number of boundary relay nodes and links, respectively. Then, the worst-case relay discovery cost becomes $C_{\mathrm{INTER}}=O\left(m_{i}m_{j}+V_{b}+E_{b}\right).$

Accordingly, the overall complexity of the WAY-CR framework for one full execution cycle can be expressed as:

(44) $$\begin{array}{cc} C_{\mathrm{WAY}-\mathrm{CR}}= & O\{n+nm+T_{\max}\left(N\log N+Nd+NC_{f}\right)\\ \; & +N_{p}L+Hn+R\;C_{\mathrm{CH}}\\ \; & +\sum_{(i,j)\in G}\left(m_{i}m_{j}+V_{b}+E_{b}\right)\}. \end{array}$$

where G is the set of neighboring GCS pairs participating in inter-GCS relay establishment. Since the metaheuristic CH selection is iterative, it constitutes the dominant computational cost in most scenarios. Hence, the practical overall complexity of WAY-CR is mainly governed by $C_{\mathrm{WAY}-\mathrm{CR}}\approx O\left(T_{\max}Nd\right),$ for moderate routing and relay-search overhead, with additional linear maintenance cost during network operation.

### 5.6. Message Complexity Analysis

The message complexity of the proposed WAY-CR framework is evaluated by counting the number of control-message transmissions required during registration, clustering, routing, maintenance, and inter-GCS relay establishment. Let *n* denote the total number of drones, *m* the number of CHs, *H* the number of maintenance rounds, *J* the total number of join/re-association events, *L* the hop length of the finally selected routing path, and $m_{i}$ the number of CHs associated with neighboring $GCS_{i}$. We count one broadcast as one logical transmission, and assume that cryptographic protection only adds constant-size authentication fields without changing the asymptotic number of exchanged messages.

**(1) Secure registration and cluster setup.** In the first stage, every drone performs secure registration with its nearest GCS before clustering. Since each registration requires only a constant number of request/response authentication packets, the registration overhead is linear in the number of drones: $M_{\mathrm{REG}}=\kappa_{\mathrm{reg}}n=O\left(n\right),$ where $\kappa_{\mathrm{reg}}$ is a constant handshake factor. The cluster-count estimation and WAY-based CH optimization are executed locally once node information is available, so they do not introduce iterative network-wide message exchanges. Thus, the optimizer increases *computational* cost, but not the *message* complexity beyond the initial state collection and final CH announcement. After CH selection, Cluster Formation requires CH advertisement and member association. If each CH broadcasts one advertisement, and each non-CH node sends one join request and receives one acknowledgment, then $M_{\mathrm{CF}}=m+2\left(n-m\right)=2n-m=O\left(n\right).$

Therefore, the total message cost for the initial intra-GCS setup is given by:

(45) $$M_{\mathrm{SETUP}}=M_{\mathrm{REG}}+M_{\mathrm{CF}}=O\left(n\right).$$

**(2) Trust-Aware Routing.** The unified trust-aware Boltzmann path-selection algorithm evaluates candidate paths using already available neighbor lists, verified relay-link information, trust, residual energy, and link stability. Hence, the scoring of candidate paths is primarily local and does not require network-wide message flooding. Once the best path is selected, the control overhead is limited to establishing or activating that route, which is proportional to the path length: $M_{\mathrm{TAR}}=O\left(L\right).$ If an inter-GCS link is not yet verified, the routing stage invokes relay discovery, whose cost is accounted for separately in $M_{\mathrm{INTER}}$.

**(3) Adaptive Cluster Maintenance.** The maintenance algorithm periodically schedules drone beacons, CH *HELLO* messages, member acknowledgments, and re-association requests for disconnected or newly arriving nodes. In one maintenance round, at most one beacon/HELLO is transmitted per drone or CH, and each active cluster member may return one acknowledgment. If $J_{h}$ denotes the number of join/re-association events during round *h*, then the message cost per round is $M_{\mathrm{MAINT}}^{\left(h\right)}=O\left(n+J_{h}\right).$ Across *H* maintenance rounds, the total maintenance overhead becomes $M_{\mathrm{MAINT}-\mathrm{total}}=O\left(Hn+J\right),$ where $J=\sum_{h=1}^{H}J_{h}$. If a CH timeout triggers re-selection, the extra communication remains bounded by the affected cluster-state refresh and announcement process, which is still linear in the number of involved drones.

**(4) Inter-GCS relay discovery and verification.** In the second stage, each local CH broadcasts a Cluster_Beacon to discover reachable CHs in adjacent GCS domains. A successful direct discovery generates one acknowledgment, while assisted or multi-hop discovery adds rebroadcast and relay-forwarding messages through boundary CMs. Let $B_{ij}$ denote the total number of boundary relay transmissions explored between neighboring $GCS_{i}$ and $GCS_{j}$. Then, the message complexity for relay discovery between these neighboring domains is $M_{\mathrm{INTER}}^{\left(i,j\right)}=O\left(m_{i}+B_{ij}\right).$ In the best case, where direct CH–CH discovery succeeds, this reduces to $O(m_{i})$. In the worst case, multi-hop controlled propagation dominates, and the overhead becomes proportional to the number of explored relay forwards. Summing over all neighboring GCS pairs in the inter-GCS graph G gives

(46) $$M_{\mathrm{INTER}}=O\;\left(\sum_{(i,j)\in G}\left(m_{i}+B_{ij}\right)\right).$$

**(5) Overall message complexity.** Combining all phases, the total message complexity of one complete execution cycle of WAY-CR is

(47) $$M_{\mathrm{WAY}-\mathrm{CR}}=O\;\left(n+n+L+Hn+J+\sum_{(i,j)\in G}\left(m_{i}+B_{ij}\right)\right),$$

which simplifies to

(48) $$M_{\mathrm{WAY}-\mathrm{CR}}=O\;\left(Hn+J+L+\sum_{(i,j)\in G}\left(m_{i}+B_{ij}\right)\right).$$

Thus, the dominant *message* overhead of WAY-CR is not produced by the metaheuristic CH optimizer itself, but by the periodic cluster-maintenance exchanges and the inter-GCS relay-discovery process. The initial registration, Cluster Formation, and route activation stages remain linear or near-linear in the network size.

## 6. Results and Discussion

This section presents and compares the results of the proposed WAY-CR framework with recent state-of-the-art FANET methods. We first evaluate the WAY optimizer against the WAA baseline, then analyze how weight settings affect clustering performance, and finally compare WAY-CR with existing approaches. The following state-of-the-art FANET methods are considered for comparative analysis: TMFCS [60], TDLS [15], SSACEER [61], SSAFANET [41], ICW [40], EESCRA [63], CSBWOA [39], and AAFSA [62]. Simulations are performed in MATLAB 2016b. The experiments focus on clustering and routing performance and on the impact of compromised nodes. The simulation settings are summarized as follows, and the simulation parameters are provided in Table 3.

### 6.1. Simulation Scenario

For experimental analysis, a FANET comprising n∈[50,150] drones is deployed in a three-dimensional region of size 2000m×2000m×500m. The GCS is located at the center of the region (1000,1000,250) and is assumed to have unlimited energy and processing capability, while each drone is initialized with the same energy level ($E_{m}=50\;J$). Drone mobility follows a smooth 3D random-walk model (SmoothRW3D) with speed uniformly bounded in [10,30]m/s and an update period of 1s per step.

To evaluate adversarial behavior, a fraction $p_{c}\in \left[0.1,0.3\right]$ of drones is designated as compromised. Trust is initialized with a threshold $T_{th}=0.5$: compromised drones are assigned low initial trust values in $\left[10^{-4},0.5\right]$, while normal drones are initialized in [0.5,1]. Communication ranges are set to $R_{uav}=250\;m$ for drone–drone (and CH–CH) links and $R_{bs}$= 1500m for CH–GCS connectivity. Link reliability is modeled using a distance and lifetime-based stability model with bounded delivery probabilities (intra-cluster: [0.85,0.98]; inter-cluster: [0.8,0.95]). The radio energy model follows the first-order model with parameters $E_{elec}=100\;\mathrm{nJ}/\mathrm{bit}$, $E_{DA}=300\;\mathrm{nJ}/\mathrm{bit}$, $\epsilon_{fs}=2\;\mathrm{pJ}/\mathrm{bit}/m^{2}$, and $\epsilon_{mp}=0.003\;\mathrm{pJ}/\mathrm{bit}/m^{4}$, using a packet size of 8192 bits. Flight-related parameters are set to $m_{U}=0.5\;\mathrm{kg}$, $w_{n}=4$ wings, and wing radius $w_{r}=0.2\;m$ (with air density $1.23\;\mathrm{kg}/m^{3}$ and $g=9.8\;m/s^{2}$) to compute a coarse flight-power proxy. Each configuration is repeated over multiple independent runs with 20 different random seeds, and results are reported as averages across runs. The complete simulation parameters are summarized in Table 3.

### 6.2. Performance Metrics

To ensure completeness and clarity, we report the *base metrics* that capture clustering and routing performance, and the *coverage/ trust-weighted* metrics that reflect connectivity and security effects, as follows.

(i) **Clustering structure and fairness:** At each time step *t*, the clustering process produces a set of CHs CH(t); thus, the number of CHs is NUMBER_OF_CHS(t)=|CH(t)|. To measure load balance among CHs, we use Jain’s fairness index over cluster sizes. Let $n_{k}\left(t\right)=\left|\left\{i:\;a_{i}\left(t\right)=ch_{k}\right\}\right|$ be the number of drones assigned to CH $ch_{k}$ (including the CH itself). Then, $\mathrm{jain}\_\mathrm{load}\left(t\right)=\frac{{\left(\sum_{k=1}^{m}n_{k}\left(t\right)\right)}^{2}}{m\sum_{k=1}^{m}n_{k}^{2}\left(t\right)},$ where m=|CH(t)|. Higher values (closer to 1) indicate more balanced clusters.
(ii) **Energy metrics:** The cumulative energy consumed by the network is computed as $\mathrm{ENERGY}\_\mathrm{USED}\_J\left(t\right)=nE_{m}-\sum_{i=1}^{n}E_{i}\left(t\right),$ where $E_{m}$ is the initial energy per drone and $E_{i}\left(t\right)$ is the residual energy of drone *i*. To incorporate coverage effects, we also report the average consumed energy per covered drone:
  (49) $$\mathrm{AvgConsEnergy}\_\mathrm{perCovered}\left(t\right)=\frac{\mathrm{ENERGY}\_\mathrm{USED}\_J(t)}{\max\left(1,N_{\mathrm{covered}}\left(t\right)\right)},$$
  where $N_{\mathrm{covered}}\left(t\right)=\left|\left\{i:\;a_{i}\left(t\right)\neq 0\right\}\right|$. For reference, AvgConsEnergy_perDrone(t)=ENERGY_USED_J(t)/n is only a normalization by *n*.
(iii) **Trust and Reliability metrics:** We evaluate the trustworthiness of the backbone by measuring the average trust of elected CHs: $\mathrm{avg}\_\mathrm{ch}\_\mathrm{trust}\left(t\right)=\frac{1}{m}\sum_{ch\in CH(t)}T_{ch}\left(t\right).$ To quantify how trust affects inter-cluster delivery, we compute trust-weighted CH-to-BS reliability as: $\mathrm{PDR}\_\mathrm{links}\_\mathrm{trust}\left(t\right)=\mathrm{avgpdr}\_\mathrm{ch}\_\mathrm{to}\_\mathrm{gcs}\left(t\right)\cdot {\bar{T}}_{\mathrm{path}}\left(t\right),$ where ${\bar{T}}_{\mathrm{path}}\left(t\right)\in \left[0,1\right]$ is the mean node trust along selected CH→BS paths (averaged over reachable paths). Hence, PDR_links_trust(t)≤avgpdr_ch_to_gcs(t). Similarly, the trust-aware global PDR applies the same coverage scaling:
  (50) $$\mathrm{PDR}\_\mathrm{global}\_\mathrm{trust}\left(t\right)=\left(\frac{\mathrm{CoveragePct}(t)}{100}\right)\cdot \mathrm{Avg}\_\mathrm{PDR}\_\mathrm{total}\_\mathrm{trust}\left(t\right).$$
  In our analysis, T_path(t) represents the *cause* metric (route trustworthiness), while PDR_links_trust(t) is the *effect* metric reflecting trust-weighted delivery.
(iv) **Backbone latency metric:** Since the focus is backbone performance, we report the CH-to-BS end-to-end delay (Avg_E2Edelay_CH), which measures latency over CH→CH→BS paths, averaged over CHs whose routes successfully reach the BS.
(v) **CH-to-GCS PDR ( avgpdr_ch_to_gcs ):** At time step *t*, each CH $ch_{k}\in CH\left(t\right)$ selects a CH→⋯→GCS path. The end-to-end PDR for $ch_{k}$ is computed as the product of hop delivery probabilities along this path (and set to 0 if the GCS is unreachable). To account for different cluster sizes, we report the cluster-size-weighted average:
  (51) $$\mathrm{avgpdr}\_\mathrm{ch}\_\mathrm{to}\_\mathrm{gcs}\left(t\right)=\sum_{k=1}^{m}w_{k}\left(t\right)\;PDR_{k}\left(t\right),\;\;\mathrm{where}\;w_{k}\left(t\right)=\frac{n_{k}\left(t\right)}{\sum_{j=1}^{m}n_{j}\left(t\right)}$$
  where $n_{k}\left(t\right)$ is the number of drones assigned to $ch_{k}$ (including the CH).

### 6.3. Performance Evaluation of the WAY Optimizer Against the WAA Baseline

To provide a comprehensive comparison between WAY and WAA, we first analyze convergence efficiency and final best-fitness behavior, and then present an ablation study to isolate the contribution of the proposed Yo-Yo operator. We use the same settings as in Section 6.1 and Table 3, with a fleet of 100 drones. Both WAY and WAA are run with a population size of 30 and an iteration budget of 100. For WAY, the Yo-Yo controller parameters are C=0.1 (vertical offset), A=1.0 (initial amplitude), $\beta_{\mathrm{damp}}=2.0$ (damping), cycles=3, ω=2π·cycles (angular frequency), and ϕ=0.0 (phase shift). Each configuration is executed for 20 independent runs. The multi-objective fitness weights correspond to a uniform configuration, where all objectives are weighted equally $\left(w_{j}=1/6\right)$.

**Figure 4:**
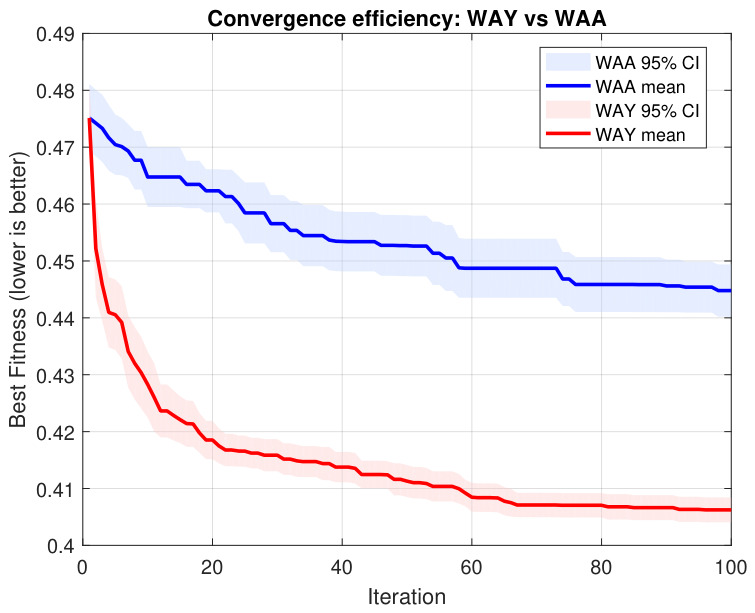
Convergence efficiency of WAY versus WAA. Bold lines show run-wise means; shaded regions denote 95% confidence intervals across runs.

Figure 4 compares the best-so-far fitness (lower is better) over iterations for WAY and the baseline WAA. WAY exhibits a steep initial descent during approximately the first 10 iterations, dropping from about 0.48 to nearly 0.42, and then enters a slower refinement phase, approaching a final plateau around 0.404. In contrast, WAA improves more gradually and levels off at a noticeably higher plateau around 0.434. The performance gap appears early and persists throughout the run, indicating that WAY reaches higher-quality solutions much faster and maintains its advantage until convergence.

**Figure 5:**
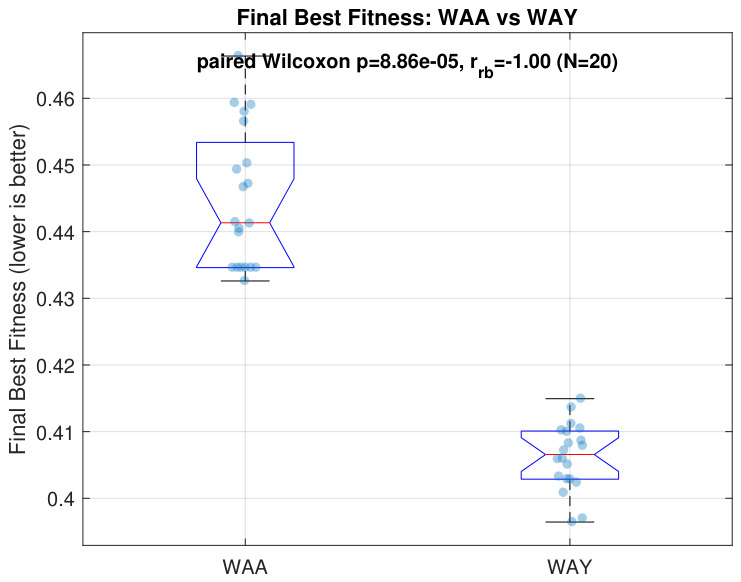
Final best fitness distributions for WAY versus WAA (lower is better). Notched boxplots show medians and their confidence intervals; dots denote paired outcomes. Paired Wilcoxon signed-rank test: $p=8.86\times 10^{-5}$, $r_{\mathrm{rb}}=-1.00$ (N=20).

Figure 5 presents the distributions of the final best fitness values for WAY and WAA across the 20 paired runs. The boxplots show that WAY consistently achieves lower final fitness (better solution quality) than WAA. The notches suggest a clear separation between the medians, while the paired outcomes further show that the improvement is systematic across runs rather than being driven by a small number of outliers. A paired Wilcoxon signed-rank test confirms that the difference is highly significant, with $p=8.86\times 10^{-5}$ and a rank-biserial effect size of $r_{\mathrm{rb}}=-1.00$. The magnitude $|r_{\mathrm{rb}}|=1$ indicates a maximal practical effect in favor of WAY, whereas the negative sign shows that WAY yields smaller (better) final fitness values than WAA in every paired run.

#### Ablation Study: WAA vs. WAY Optimizer

To isolate the contribution of the proposed Yo-Yo operator, we compared the baseline WAA against its Yo-Yo-enhanced variant, denoted as WAY, under matched scenarios. Table 4 reports the results separately for n=50, 100, and 150, together with a pooled summary over all matched runs. The results show that the Yo-Yo operator consistently preserves full coverage while making the clustering structure substantially reduced and more energy-efficient. In particular, the number of CHs is reduced by 77.9%, 83.9%, and 86.5% for n=50, 100, and 150, respectively, with a pooled reduction of 84.2%. Similarly, total energy consumption decreases by 34.8%, 43.7%, and 48.1%, yielding a pooled reduction of 44.3%. At the same time, WAY improves CH trust, path trust, trust-aware link reliability, Jain load fairness, and trust-weighted total PDR across all network sizes, confirming that the Yo-Yo operator consistently enhances the trust-oriented behavior of the FANET.

The main trade-off is that the more compact CH structure comes at the cost of higher delay. Although the raw total PDR decreases only slightly across all sizes, from approximately 0.867-0.873 under WAA to 0.860-0.861 under WAY. In addition, the average node-level end-to-end delay increases by about 25.8% to 28.7%. Overall, the ablation confirms that the Yo-Yo operator is the key component responsible for transforming WAA into a more selective, balanced, and trust-aware FANET solution, while introducing a clear trade-off in CH persistence and node-level latency.

### 6.4. Impact of Weight Selection on Clustering Performance

To investigate the sensitivity of the proposed clustering scheme to the multi-objective fitness weights, we consider seven weight configurations for the six objectives $\left[f_{E},f_{CD},f_{DB},f_{LB},f_{SV},f_{OT}\right]$. Cases 1–6 are single-dominant settings, in which one objective is assigned a weight of 0.5 while the remaining five objectives each receive 0.1. These correspond to energy-dominant (Case 1), intra-cluster-distance-dominant (Case 2), distance-to-BS-dominant (Case 3), load-balancing-dominant (Case 4), speed-variation-dominant (Case 5), and trust-dominant (Case 6) configurations. Case 7 is the uniform setting, in which all objectives are weighted equally $\left(w_{j}=1/6\right)$. Together, these cases span a representative range of design priorities and allow us to assess how emphasizing a single objective, or weighting all objectives uniformly, affects cluster-head selection and network performance.

Among these seven configurations, we focus on three representative cases:

1. The intra-cluster-distance-dominant case, w=[0.1,0.5,0.1,0.1,0.1,0.1];
2. The load-balancing-dominant case, w=[0.1,0.1,0.1,0.5,0.1,0.1];
3. The uniform configuration, $w_{j}=1/6\;\mathrm{for}\;\mathrm{all}\;j.$

In the $f_{CD}$-dominant case, the optimizer selects on average 12.3 CHs, yielding the lowest average energy consumption (257.7 units) and the highest average packet delivery ratio, ${P\bar{D}R}_{\mathrm{total}}=0.8692,$ but at the cost of lower fairness, with Jain’s index equal to 0.8846. This suggests that prioritizing intra-cluster distance produces many small clusters with shorter communication links, which improves energy efficiency and reliability, but makes the distribution of members across CHs less uniform.

In contrast, the $f_{LB}$-dominant setting produces a smaller number of CHs on average (8.3), with slightly higher energy consumption (290.6 units), but significantly improves load fairness (Jain’s index =0.9310) while still maintaining a high average PDR (0.8669) and the best observed PDR over all runs, ${\mathrm{PDR}}_{\mathrm{total},\mathrm{best}}=0.8752.$ The uniform-weight configuration behaves as a balanced compromise between these two extremes. It yields 8.6 CHs on average, intermediate energy consumption (279.4 units), and robust performance in both reliability, ${P\bar{D}R}_{\mathrm{total}}=0.8676,\;{\mathrm{PDR}}_{\mathrm{total},\mathrm{best}}=0.8748,$ and fairness (Jain’s index =0.9175).

**Figure 6:**
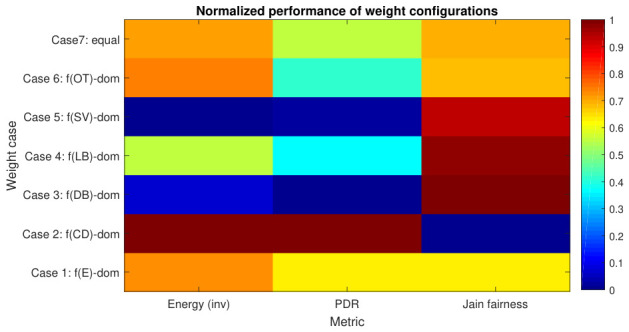
Normalized performance results of the seven weight configurations in terms of inverted energy consumption, average PDR, and Jain’s load-fairness index. Each metric is scaled to [0,1], with brighter colors indicating better performance.

Figure 6 visually confirms the same trends. In particular, the $f_{CD}$-dominant configuration achieves the strongest energy–PDR combination but with weaker fairness, whereas the $f_{DB}$- and $f_{LB}$-dominant configurations provide the highest fairness. The equal-weight setting remains the most balanced overall configuration. By contrast, the $f_{SV}$-dominant case performs poorly in both energy and PDR. Since all drones have similar speeds in our scenarios, assigning a high weight to the speed-variation objective $f_{SV}$ does not yield meaningful gains in reliability or fairness. Therefore, in the remainder of the experiments, we adopt the uniform setting $w_{j}=1/6$ for all *j* as the main baseline.

### 6.5. Comparison of WAY-CR with Existing Approaches

This subsection presents the comparative analysis results of **WAY-CR** against existing approaches, using the performance metrics given in Section 6.2. We first analyze and compare the clustering and routing performance, and then examine the impact of compromised nodes on network robustness.

#### 6.5.1. Analysis: Clustering and Routing Performance

This section discusses the clustering and routing performance of the WAY and the baseline methods in terms of multiple metrics. We infer the following from the experimental results. As the number of drones increases from 50 to 150, all baseline methods elect a larger number of CHs, which expands the backbone and increases clustering/routing overhead. In contrast, WAY-CR consistently forms the smallest CH set across all network sizes, indicating more compact and scalable backbone formation.

**Figure 7:**
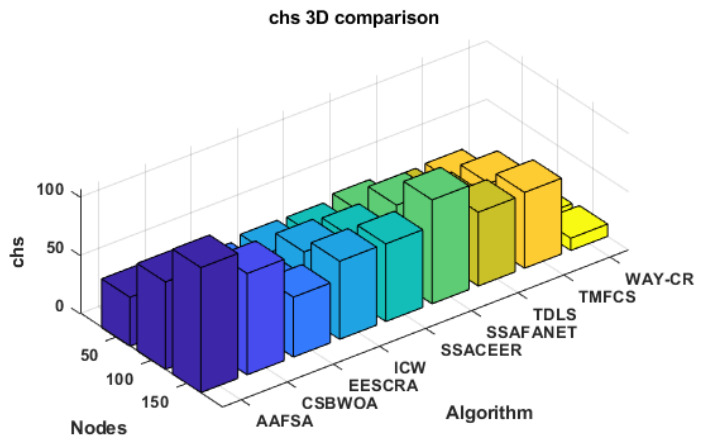
Number of CHs vs. number of drones (n=50,100,150).

In terms of load balance, WAY-CR achieves the highest and most stable Jain index (0.909 for n=50,100,150), showing that it maintains well-balanced cluster sizes as the network scales. Most baselines exhibit decreasing fairness with larger networks (e.g., TMFCS: 0.7989→0.7203, AAFSA: 0.8373→0.6544), while EESCRA becomes highly imbalanced (0.2490→0.0492). These results confirm that WAY-CR improves scalability by limiting backbone size and preserving cluster-size fairness under increasing network density. The results are discussed in detail, as follows:

**(i) Scalability: number of CHs:** Figure 7 compares the number of elected CHs under different network sizes (n=50,100,150). As *n* increases, all baseline methods tend to elect more CHs, which enlarges the backbone and increases clustering and routing overhead. In contrast, WAY-CR consistently produces the smallest CH set across all node densities, indicating a more compact backbone formation and reduced control overhead. Notably, methods such as SSAFANET and AAFSA exhibit the highest CH counts at large *n*, implying higher inter-cluster coordination cost and potentially more frequent backbone maintenance.

**Figure 8:**
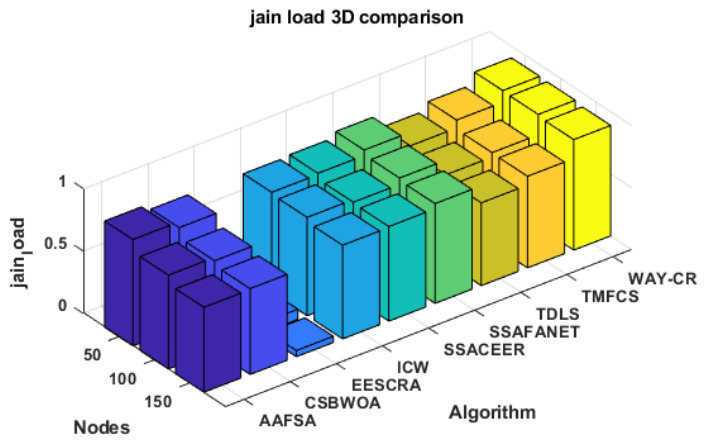
Jain load fairness vs. number of drones (n=50,100,150).

**(ii) Load balance: Jain fairness of cluster sizes:** Figure 8 reports Jain’s load fairness index, which captures how evenly drones are distributed among CHs. WAY-CR achieves the highest and most stable fairness across all tested network sizes (Jain =0.909 for n=50,100,150), indicating that it maintains balanced cluster sizes even as the network scales. In contrast, most baselines show a monotonic decrease in fairness with increasing *n* (e.g., TMFCS: 0.7989→0.7203 and AAFSA: 0.8373→0.6544), reflecting increasing load imbalance at higher densities. EESCRA exhibits the lowest fairness values (0.2490→0.0492), which indicates highly uneven clustering and suggests that a small subset of CHs becomes overloaded. Overall, these results show that WAY-CR improves scalability by limiting backbone size while maintaining superior load balancing.

**Figure 9:**
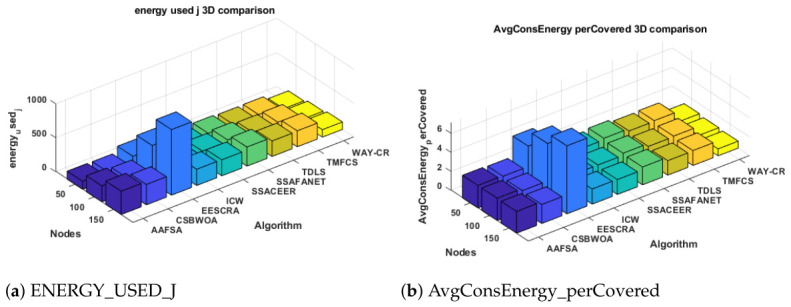
Energy efficiency under different network sizes (n=50,100,150).

**(iii) Energy consumption:** Figure 9a reports the cumulative energy consumption (ENERGY_USED_J) for different network sizes (n=50,100,150). As expected, energy consumption increases with *n* for all methods due to the higher communication and processing load. However, WAY-CR consistently achieves the lowest energy usage across all scenarios (23.70 J, 40.18 J, and 54.15 J for n=50,100,150, respectively). Compared to the most energy-efficient baseline (TDLS), WAY-CR reduces energy consumption by approximately 51–52% across all network sizes (e.g., at n=150: 54.15 J vs. 112.20 J). In contrast, EESCRA incurs the highest energy cost, which becomes increasingly severe as the network grows (e.g., 474.28 J at n=150), indicating substantial overhead and inefficient load distribution. The energy gains of WAY-CR are consistent with its compact backbone formation (fewer CHs) and balanced clustering, which reduce unnecessary control and relay operations.

**(iv) Average consumed energy per covered drone:** Figure 9b shows the results in terms of AvgConsEnergy_perCovered, defined as the consumed energy normalized by the number of covered drones. In these experiments, all drones remain covered (i.e., $N_{\mathrm{covered}}\approx n$); therefore, AvgConsEnergy_perCovered≈ENERGY_USED_J/n. WAY-CR again achieves the lowest values (0.474, 0.402, and 0.361 J per drone for n=50,100,150), which is about 51–52% lower than the best baseline (TDLS) at each network size. EESCRA shows the highest normalized cost, rising sharply with *n* (2.23, 2.96, and 3.51 J per drone), which reflects its high absolute energy consumption under scaling.

**Figure 10:**
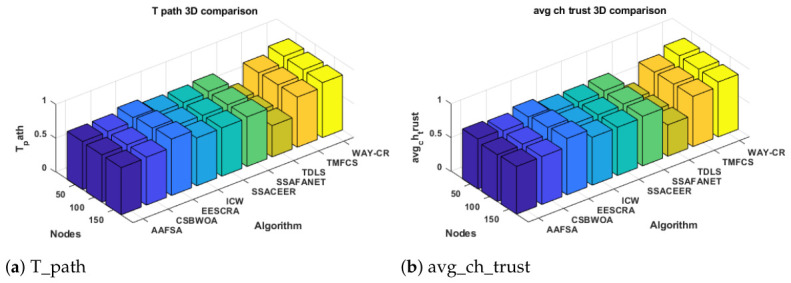
Trust cause metrics versus number of drones (n=50,100,150).

**(v) Route and CH trust (cause metrics):** Figure 10a reports the route trust metric T_path, while Figure 10b reports the average trust of the elected CHs ( avg_ch_trust). WAY-CR maintains consistently high trust values across all network sizes (n=50,100,150), with only a mild decrease as the network scales (e.g., T_path: 0.815→0.802→0.791). This indicates that the proposed clustering and backbone selection favor trustworthy relays even in denser deployments. Although EESCRA attains higher trust values at larger *n* (e.g., T_path reaches 0.838 at n=150), this does not necessarily imply better overall performance because trust must be considered jointly with coverage, energy cost, and clustering balance.

**Figure 11:**
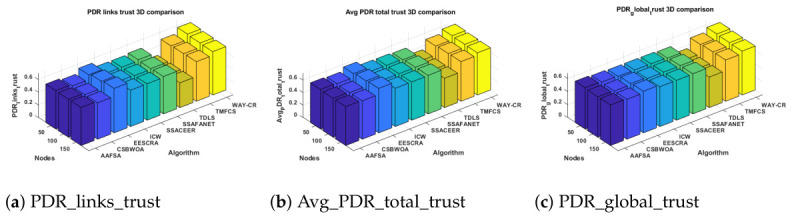
Trust effect metrics versus number of drones (n=50,100,150).

**(vi) Trust-weighted backbone reliability (effect metric):** Figure 11a represents the CH-to-GCS delivery probability weighted by path trust (PDR_links_trust). As expected, the results follow the same ordering as the underlying trust metrics: methods with higher T_path generally achieve higher trust-weighted backbone reliability. WAY-CR provides strong and stable backbone reliability (approximately 0.689→0.677→0.666 as *n* increases), outperforming most baselines. EESCRA becomes higher at larger *n* (up to 0.708 at n=150), reflecting its high trust in the selected backbone paths.

**(vii) Trust-aware total and global reliability:** Figure 11b reports Avg_PDR_total_trust, which combines trust-weighted inter-cluster delivery with intra-cluster delivery, while Figure 11c further applies coverage scaling to obtain PDR_global_trust. EESCRA yields the highest values as *n* increases (e.g., 0.683→0.701→0.716). However, after accounting for coverage, WAY-CR achieves the best global trust-aware performance (e.g., PDR_global_trust: 0.672→0.664→0.658), while EESCRA drops substantially (e.g., 0.615→0.631→0.644), indicating that its higher trust-weighted reliability is partly offset by reduced coverage. This confirms that PDR_global_trust provides a more conservative and system-level view by penalizing unreachable or uncovered drones.

**Figure 12:**
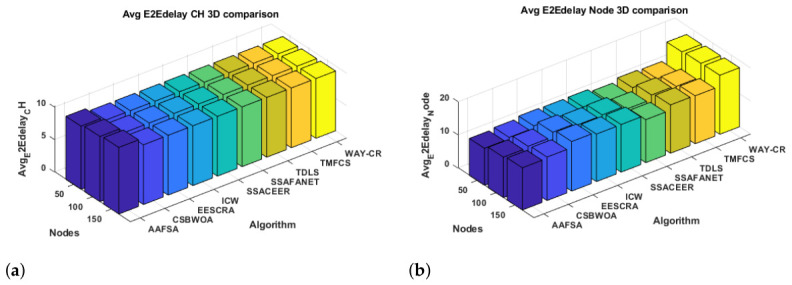
End-to-end delay versus number of drones (n=50,100,150). (**a**) Average CH-to-GCS end-to-end delay (Avg_E2Edelay_CH). (**b**) Access + backbone end-to-end delay (Avg_E2Edelay_Node).

**(viii) Backbone latency (CH-to-BS delay):** Figure 12a reports the average CH-to-BS end-to-end delay, given by Avg_E2Edelay_CH, for different network sizes (n=50,100,150). The results show that WAY-CR achieves a stable backbone delay of approximately 9.19 ms across all tested network sizes, which is comparable to most baseline methods (TMFCS, TDLS, SSACEER, SSAFANET, ICW, EESCRA, and CSBWOA). In contrast, AAFSA exhibits consistently higher delay that increases with network size (from 9.67 ms at n=50 to 10.11 ms at n=150), suggesting longer CH-to-BS routes and/or higher hop counts. Overall, the WAY-CR maintains low backbone latency while scaling to larger networks.

**(ix) Access + backbone delay (node-to-BS latency):** Although our main latency KPI targets the backbone (CH→BS), we additionally report the *access + backbone* end-to-end delay, Avg_E2Edelay_Node, in Figure 12b. This metric includes both the intra-cluster access hop (CM→CH) and the inter-cluster backbone path (CH→⋯→BS). WAY-CR shows higher node-to-BS delay across all network sizes (approximately 17.37→17.65→17.77 ms for n=50,100,150), while most baselines remain in the range of ∼11–15 ms. This outcome is consistent with WAY-CR’s compact clustering (fewer CHs): larger clusters increase the average CM→CH distance, which raises the access component of latency. Importantly, the backbone delay Avg_E2Edelay_CH remains low and comparable to other methods; therefore, the increase in Avg_E2Edelay_Node primarily reflects an access-to-backbone trade-off rather than inefficient backbone routing.

Although WAY-CR yields a higher Avg_E2Edelay_Node than several baselines, the absolute delay remains low (approximately 17–18 ms across all network sizes), which is acceptable for typical FANET monitoring and coordination applications. This observation is also consistent with the simulator delay model. In our implementation, the per-hop delay is dominated by the transmission and processing components: $D_{\mathrm{hop}}=\frac{L}{R_{b}}+d_{\mathrm{proc}}+\frac{d}{c},$ where L=8192 bits, $R_{b}=10^{6}$ bps, and $d_{\mathrm{proc}}=1$ ms, while the propagation term d/c is negligible (microseconds). Therefore, the transmission time is $L/R_{b}=8192/10^{6}\approx 8.19$ ms and the per-hop processing delay adds ≈1 ms, giving $D_{\mathrm{hop}}\approx 9.19$ ms. Consequently, the observed WAY-CR node-to-BS delay of ∼17.5 ms corresponds to roughly two hops on average (CM→CH plus ≈ one CH→BS backbone hop), indicating reasonable routing behavior rather than excessive latency. The slightly higher Avg_E2Edelay_Node is primarily attributed to the access component (CM→CH), since WAY-CR forms fewer CHs and hence larger clusters. Importantly, the backbone delay Avg_E2Edelay_CH remains comparable to other methods; therefore, the node-to-BS latency reflects a modest access-to-backbone trade-off in exchange for improved energy efficiency, reduced clustering overhead, and stronger trust-aware reliability.

**Figure 13:**
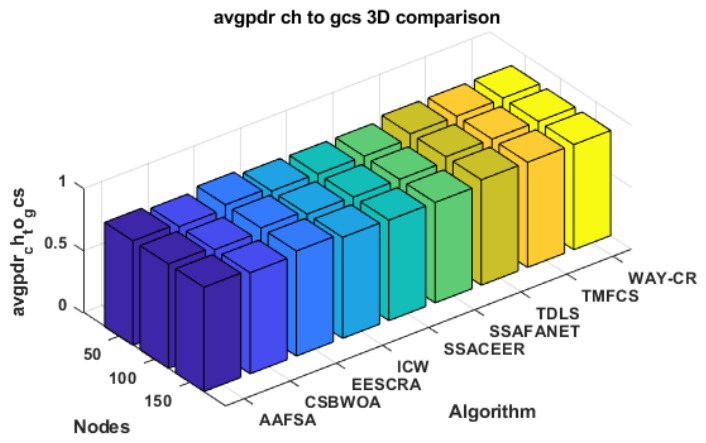
Average CH-to-GCS PDR (avgpdr_ch_to_gcs) versus number of drones (n=50,100,150).

**(x) Backbone delivery reliability (CH-to-GCS PDR):** Figure 13 presents the average CH-to-GCS delivery reliability (avgpdr_ch_to_gcs) over the selected backbone paths. Overall, this metric remains high and stable across all methods (approximately 0.80–0.846) and changes only slightly as the network scales from n=50 to n=150. TDLS and EESCRA achieve the highest CH-to-GCS PDR (e.g., TDLS: 0.8433→0.8450→0.8458), while SSACEER and SSAFANET show the lowest values (around 0.805). WAY-CR also maintains strong backbone reliability (about 0.845→0.844→0.843), indicating robust CH-to-GCS communication. Since this metric captures only backbone link delivery, we further analyze coverage- and trust-weighted metrics to reflect end-to-end system performance under partial connectivity and adversarial behavior.

As summarized in Table 5, WAY-CR consistently achieves the best energy efficiency and clustering overhead across all node counts, while maintaining strong trust-aware delivery. Although minor negative gains are observed at n=150 for PDR_links_trust (0.6814/0.7002, −2.68%) and avg_ch_trust (0.8085/0.8295, −2.53%), these differences are small and do not indicate a meaningful degradation in backbone reliability. Importantly, the obtained trust values remain above the adopted trust acceptance threshold, confirming that WAY-CR preserves a trustworthy backbone even when it is marginally outperformed by the strongest baseline in a single operating point.

#### 6.5.2. Analysis: Impact of Compromised Nodes

This section stress-tests the robustness of the proposed WAY-CR against insider compromise by increasing the ratio of compromised drones from 10% to 30%. Since several KPIs are trust-weighted or coverage-weighted (e.g., trust-aware delivery), results are interpreted using a *cause/effect* perspective: backbone trustworthiness (*cause*) versus trust-aware delivery performance (*effect*). Studying such adversarial conditions is critical because compromised nodes can mislead clustering decisions, degrade routing reliability, and reduce overall network performance.

**Figure 14:**
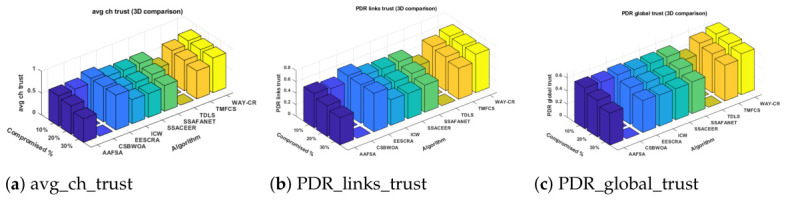
Impact of compromised nodes (10–30%) on backbone trustworthiness and trust-aware delivery.

**(i) Backbone trustworthiness (cause metrics):** The above stability is consistent with the trust properties of the selected backbone. Figure 14a confirms that WAY-CR sustains high cluster-head trust as compromise increases, remaining within 0.8150→0.7930. This sustained CH trust directly supports stable trust-aware forwarding and prevents compromised nodes from dominating backbone decisions.

**(ii) Trust-aware delivery (effect metrics):** For CH-to-GCS trust-weighted forwarding, Figure 14b shows that PDR_links_trust remains highly stable for WAY-CR (0.6875→0.6702 from 10% to 30%, i.e., only ≈2.52% reduction). At 10% and 20%, WAY-CR is the best performer, and at 30% it is effectively tied with the best baseline (EESCRA), with a negligible difference of 0.00055 (−0.08% relative).

**(iii) Raw vs. trust-aware delivery:** Figure 14c shows that WAY-CR achieves the highest trust-aware global delivery at all compromise levels. Specifically, PDR_global_trust decreases from 0.6758 at 10% to 0.6232 at 30%, corresponding to a relative drop of only ≈7.78%. In contrast, most competitors experience a substantially larger degradation of ≈21.4–23.6%, while the strongest baseline (TMFCS) still drops by ≈13.47%. This indicates that WAY-CR preserves trusted connectivity and delivery even as adversarial presence increases. Although WAY-CR does not always maximize the *raw* delivery PDR_global (which ignores trust), it consistently dominates trust-aware delivery PDR_global_trust under all compromise levels (Figure 14c). This gap reflects the design objective of WAY-CR: prioritizing delivery through *trusted* relays rather than maximizing throughput through potentially compromised or low-trust paths.

**Figure 15:**
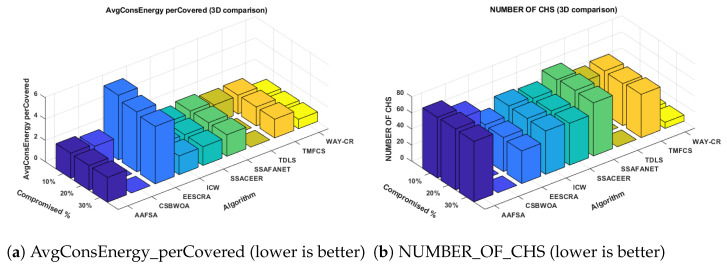
Impact of compromised nodes on energy consumption and clustering overhead.

**(iv) Energy efficiency and clustering overhead:** Figure 15a reports the energy consumed per *covered* drone. WAY-CR is consistently the most energy-efficient method, achieving AvgConsEnergy_perCovered=0.8184→0.8845 as compromise increases. Compared to the best baseline (TDLS) at each operating point, this corresponds to an energy reduction of approximately 48.83%−51.76% (Table 6), demonstrating that WAY-CR improves efficiency even under increasing adversarial pressure. In addition, WAY-CR substantially reduces clustering overhead. Figure 15b shows that WAY-CR uses only 8.25→7.55 CHs on average, whereas the strongest competing baseline still requires 40.4−43.1 CHs. This corresponds to about 80.86%−81.31% fewer CHs, lowering control overhead and reducing contention and attack exposure within the backbone.

**Figure 16:**
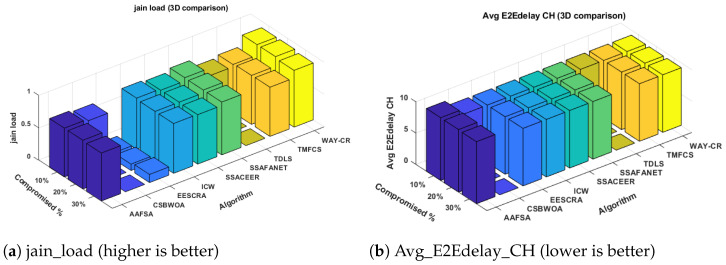
Impact of compromised nodes on fairness and backbone latency.

**(v) Fairness and latency:** Balanced load is crucial under compromise because overloaded CHs become bottlenecks and high-value attack targets. Figure 16a shows that WAY-CR achieves the highest fairness, with jain_load≈0.900 at 10–20% and 0.8746 at 30%, improving by ≈7.60%–10.81% over the best baseline (Table 6). Meanwhile, backbone latency remains essentially unchanged across methods; Figure 16b confirms that Avg_E2Edelay_CH≈9.194 for WAY-CR, with only negligible gains versus the best baseline (<0.01%).

**Overall analysis:** Table 6 summarizes the mean performance under 10%, 20%, and 30% compromised nodes. As the compromised-node ratio increases from 10% to 30%, most methods exhibit a clear decline in backbone trust (avg_ch_trust), which propagates to trust-weighted delivery performance (PDR_links_trust and PDR_global_trust). Delay remains nearly constant for all methods (Avg_E2Edelay_CH≈9.195), except AAFSA, which retains higher latency. The most discriminative trends, therefore, appear in trust-weighted delivery, clustering overhead, fairness, and energy efficiency. WAY-CR is the least sensitive to compromise while maintaining the strongest performance overall. PDR_global_trust drops only from 0.6758 to 0.6232 (−7.78%), and PDR_links_trust remains stable (0.6875→0.6702, −2.52%), supported by consistently high avg_ch_trust (0.8150→0.7930, −2.70%). Energy efficiency remains the best across all settings (AvgConsEnergy_perCovered=0.8184→0.8845), while clustering overhead decreases further under stronger attacks (NUMBER_OF_CHS=8.25→7.55, −8.48%). Fairness remains the highest (jain_load=0.9001→0.8746). TMFCS provides the strongest baseline for trust-aware global delivery, but degrades more rapidly than WAY-CR: PDR_global_trust decreases from 0.6293 to 0.5446 (−13.47%) and PDR_links_trust from 0.6189 to 0.5403 (−12.71%), consistent with a decline in avg_ch_trust (0.7345→0.6400, −12.87%). Energy remains nearly unchanged, and NUMBER_OF_CHS slightly increases. EESCRA maintains very stable backbone trust and link-trust delivery: avg_ch_trust=0.8085→0.7960 (−1.55%) and PDR_links_trust=0.6808→0.6708 (−1.48%). However, its PDR_global_trust still drops sharply (0.6288→0.4865, −22.63%), and its energy consumption is consistently the highest among all methods (AvgConsEnergy_perCovered≈5.38−6.06). The fairness index rises with compromise, yet remains low in absolute terms. Other methods exhibit strong sensitivity to increasing compromise, with large trust and delivery degradation. For instance, PDR_global_trust drops by −21.37% (SSAFANET), −22.55% (SSACEER), −22.49% (ICW), and −23.61% (CSBWOA), accompanied by comparable declines in PDR_links_trust and avg_ch_trust. Their energy and CH counts remain relatively stable, but none approaches the efficiency or trust-aware delivery of WAY-CR. However, TDLS remains almost constant across compromise levels in trust-aware delivery (PDR_global_trust≈0.466), but its absolute trust-aware performance is significantly lower than all other methods. TDLS is the strongest baseline for energy among the competitors, yet it remains far less efficient than WAY-CR. AAFSA experiences strong delivery degradation under compromise (PDR_global_trust:0.6215→0.4864, −21.74%), while also incurring the highest delay (Avg_E2Edelay_CH≈10). Its CH count remains very high (≈75), leading to persistent overhead under attack.

### 6.6. Discussion Summary

This section summarizes why the proposed WAY-CR achieves higher performance than state-of-the-art FANET solutions, including TMFCS [60], TDLS [15], SSACEER [61], SSAFANET [41], ICW [40], EESCRA [63], CSBWOA [39], and AAFSA [62].

As reported in Table 5 and Table 6, WAY-CR improves trusted delivery while simultaneously reducing control overhead and energy cost. In particular, WAY-CR increases PDR_global_trust by up to 14.44% under severe compromise, improves load fairness (Jain’s index) by approximately 5.7%–10.8%, reduces AvgConsEnergy_perCovered by about 49–52%, and decreases the number of CHs by approximately 79.5%–81.6%, without increasing end-to-end delay. These gains stem from three main design choices: *(i)* a unified multi-criteria clustering fitness that jointly considers energy, trust, mobility stability, distance efficiency, and fairness, *(ii)* Trust-Aware Boltzmann path selection that enables adaptive and reliable routing under dynamic conditions, and *(iii)* WAY metaheuristic optimizer to select stable CHs while balancing exploration and exploitation.

TMFCS relies on hybrid trust (message and interaction trusts) and trust-based CH selection; however, its trust updating process is mainly reactive, and it lacks strong joint optimization for mobility stability and fairness. As a result, its global trusted delivery degrades faster as attacks intensify, whereas WAY-CR sustains a higher PDR_global_trust (Table 6). Similarly, TDLS provides trust-based leader selection, but frequent leader and cluster reconfiguration under mobility increases control overhead and energy consumption.

In contrast, WAY-CR explicitly controls CH population and applies adaptive maintenance, leading to consistently lower AvgConsEnergy_perCovered across all compromise levels (Table 5 and Table 6). EESCRA is highly competitive at link-level trust forwarding, which explains why PDR_links_trust and avg_ch_trust can be close to (or slightly higher than) WAY-CR in dense settings. However, EESCRA typically forms a significantly larger number of CHs to maintain connectivity, which increases overhead and reduces global efficiency. WAY-CR mitigates this limitation through CH-count targeting and fairness-aware clustering, achieving fewer CHs and better scalability (Table 5). SSAFANET, which integrates trust and energy using SSA, does not explicitly enforce fairness (Jain’s index) or CH-count constraints. This may result in unbalanced CH workloads and reduced stability in larger networks. By contrast, WAY-CR directly optimizes fairness and load distribution, producing consistently higher jain_load as the network size increases (Table 5). SSACEER, CSBWOA, and ICW mainly focus on energy- or stability-oriented clustering with geographic or conventional routing. However, their routing decisions are not fully trust-integrated under adversarial conditions. Consequently, these methods may still select routes that traverse compromised relays, which reduces trusted delivery in post-disaster scenarios. AAFSA improves CH selection through fish swarm intelligence, but it lacks explicit global controls for fairness, CH population targeting, and coverage-aware stability, making it more sensitive to dense and high-mobility scenarios.

WAY-CR addresses these limitations by embedding trust into both CH selection and route selection, while maintaining comparable delay (Table 5 and Table 6). WAY-CR strengthens exploration-exploitation through the Yo-Yo Motion Operator and uses a unified fitness incorporating fairness and stability, which improves clustering balance and trusted delivery at scale (Table 5). Overall, the baseline methods typically suffer from one or more limitations: incomplete joint optimization across clustering and routing, weak fairness and CH-count control, and limited trust integration under high compromise. WAY-CR overcomes these limitations using unified optimization, trust-aware probabilistic routing, and adaptive maintenance, which explains its consistent gains in trusted delivery, load balancing, CH reduction, and energy efficiency (Table 5 and Table 6).

## 7. Conclusions

This paper presented WAY-CR, a trust-aware and mobility-adaptive framework for drone-assisted communication recovery in post-disaster FANET environments. In Stage 1, WAY-CR performs intra-GCS clustering, routing, and maintenance. An enhanced WAY optimizer is employed to select stable CHs using a unified multi-criteria objective. This stage organizes drones into authenticated clusters and establishes a trusted intra-GCS backbone, enabling secure data exchange between CMs under their associated GCS domains. When network fragmentation or GCS disconnection occurs, Stage 2 restores mission-level connectivity by enabling secure inter-GCS communication through authenticated CH–CM relay links. Furthermore, WAY-CR employs a trust-aware Boltzmann-based routing strategy to support both intra-GCS and inter-GCS routing decisions, improving reliability under dynamic mobility and adversarial conditions. Extensive simulations against several state-of-the-art FANET clustering and routing baselines demonstrate that WAY-CR consistently achieves superior performance across varying network sizes and compromise levels. In particular, WAY-CR consistently outperforms the best baseline at each setting, improving PDR_global_trust by 5.19–14.44% and load fairness by 5.73–10.81%, while reducing energy consumption per covered drone by 48.83–51.96% and decreasing the number of CHs by 79.55–81.61%, without incurring any delay penalty.

### Limitations and Future Work

Despite the promising performance of WAY-CR, several limitations should be acknowledged. First, this work does not provide implementation-level measurements of the computational, memory, or energy overhead introduced by Paillier homomorphic encryption on resource-constrained drone platforms. Although related encryption mechanisms have been investigated in prior drone-oriented studies [69,70], hardware-level validation of lightweight secure deployment is still needed.

Second, the evaluation is based on simulation rather than real-world flight experiments. The simulation setup was designed to reflect practical post-disaster conditions by incorporating drone mobility, energy constraints, communication limitations, dynamic trust assessment, and authentication within a secure and adaptive FANET framework. However, real-world drone experiments are still required to validate the framework under physical channel impairments, hardware limitations, and operational uncertainties.

Third, WAY-CR integrates several functional modules, including clustering optimization, trust evaluation, secure communication, and adaptive routing. In practice, the framework is modular, and its components can be simplified or introduced incrementally according to mission requirements and hardware capabilities. Prototype-oriented validation and reproducibility-focused implementation therefore remain part of ongoing and future work.

Fourth, the Yo-Yo operator consistently improves energy efficiency, trust-related metrics, and load fairness, but these gains come with a trade-off in the form of higher node-level end-to-end delay. In addition, the Yo-Yo operator was evaluated only within the WAA framework; therefore, its applicability to other FANET clustering and routing strategies remains an open question.

Future work will focus on deployment-aware decision-making for disaster environments, including adaptive placement strategies for recovery and coordination sites under dynamic constraints. We also plan to extend WAY-CR through tighter IoT integration in order to improve situational awareness, coordination efficiency, and network adaptability in heterogeneous disaster-response scenarios.

## Figures and Tables

**Table 1:** Comparative summary of trust-aware and optimization-based clustering and routing methods in FANETs

Method (Ref)	Trust Mechanism	Clustering Strategy	Routing Focus
TDLS-FANET ( [15])	Hybrid trust (QoS, social trust, fitness score)	Dynamic leader (CH) selection	Reduce delay and energy, improve PDR via dynamic leader routing.
TEM-FANET ( [48])	Blockchain-enabled direct/indirect trust	Flat trust-based network organization	Trust-based secure routing that selects high-trust paths.
CLARA ( [50])	None (no explicit trust model)	Multi-phase adaptive clustering using learning automata	Energy-aware and resilient route formation.
FBTMD ( [47])	Fuzzy logic with direct and indirect trust	Trust-based CH validation	Authentication process isolates malicious drones.
MWCRSF ( [53])	None (mobility and connectivity metrics only)	Mobility-aware clustering using SSA	Skyline-filtered routing to improve stability and connectivity.
ICRP ( [14])	None (no explicit trust model)	Hybrid relay-assisted routing (ACO + Physarum-inspired)	Load-aware and predictive routing with adaptive path updates.
PICA ( [59])	None (no explicit trust model)	Physarum-inspired distributed clustering	Stable clusters with reduced re-clustering frequency.
ICBM-UAV [57]	None (no explicit trust model)	Intelligent CH assignment for search-and-rescue missions	Victim-oriented communication with reduced latency and energy usage.
HMGOC [58]	None (no explicit trust model)	Hybrid MGO and Jaya-based clustering	Latency-aware and load-balanced routing using Bayesian inference.
SCFS [46]	Dynamic fuzzy trust evaluation	Adaptive re-clustering based on trust variations	Trust recovery and malicious node isolation.
TMFCS [60]	Direct and indirect trust using behavioral and message-level parameters	Fitness-based k-means density clustering with trust- and energy-aware CH selection	Trust-based CM-CH-GS data delivery without full end-to-end multi-hop routing.
SSAFANET [41]	Scalar trust-based classification: malicious or distrusted or normal	SSA-based clustering with multi-parameter CH fitness and CH reselection for stability	Energy- and distance-aware CM-CH-GS multi-hop routing (no explicit secure routing design).
ICW [40]	None (no explicit trust model; energy and mobility metrics only)	WOA-based clustering with adaptive hello intervals (residual energy, link lifetime, neighbor degree, and average neighbor distance)	Intra-cluster greedy routing and inter-cluster multi-hop CH-CH routing based on energy, distance, and link lifetime.
SSACEER [61]	None (no explicit trust or security model; energy and mobility-based metrics only)	SSA-based clustering with mobility-aware CH selection	Position-based greedy routing (LQDR) toward the BS.
AAFSA [62]	Direct and indirect trust	CH election using artificial fish swarm fitness (link capacity, energy, density)	Trust-aware multi-hop routing via ITOPSIS-based path ranking.
EESCRA [63]	Direct trust; low-trust drones marked malicious and excluded	Coverage-based clustering with trust- and energy-aware CH selection	Secure and energy-efficient CM-CH-CH multi-hop routing using path ranking via a composite metric.
AODV and OLSR [42]	No explicit trust or security mechanism	No explicit clustering optimization; flat routing comparison	Baseline FANET comparison of AODV and OLSR, where AODV improved PDR/throughput, and OLSR reduced delay/jitter.
WAY-CR (Proposed)	Multi-dimensional trust with fitness amplification	WAY-based adaptive clustering (energy–trust–mobility)	Trust-aware Boltzmann routing with UCB temperature tuning

**Table 2:** Comparison: WAA versus proposed WAY algorithm.

Aspect	WAA (Original)	WAY (Proposed)
Phase Control	Random selector $k_{1}$ with abrupt switching	Continuous sinusoidal control $k_{1}\left(t\right)$ (Yo-Yo motion)
Exploration Methods	Lévy flight or random reinitialization based on $k_{3}$	Same, triggered during $k_{1}\left(t\right)<0.5$ (smooth phase)
Exploitation Modes	3 random strategies selected via $k_{2}$	Single unified update toward weighted guidance point
Stability	Non-deterministic behavior due to 3 random selectors	Deterministic motion controller; more stable updates
Oscillation Decay	No decay (constant randomness)	Damped oscillation via $e^{-\beta_{\mathrm{damp}}t/T}$
Exploration-Exploitation Balance	Hard switching with probability	Naturally alternating via sine-based Yo-Yo operator
Complexity	O(N·d·T)	O(N·d·T)

**Table 3:** Simulation parameters.

Parameter	Specification
Network dimensions	2000×2000×500 m
Simulation duration	120 s
Mobility model	SmoothRW3D [64]
Drone speed range	10–30 m/s
Number of drones	n∈[50,150]
Drone–drone / CH–CH range	$R_{uav}=250$ m
CH–BS range	$R_{bs}=1500$ m
Minimum drone spacing	10 m
Initial energy per drone	$E_{m}=50$ J
Energy threshold (unintentional loss)	$E_{th}=0.1E_{m}$
Packet size	8192 bits
Electronics energy	$E_{elec}=100$ nJ/bit
Data aggregation energy	$E_{DA}=300$ nJ/bit
Free-space amplifier	$\epsilon_{fs}=2$ pJ/bit/m^2^
Multipath amplifier	$\epsilon_{mp}=0.003$ pJ/bit/m^4^
Air density	1.23 kg/m^3^
Gravitational acceleration	9.8 m/s^2^
Data rate	1 Mbps
Processing delay per hop	1 ms
Compromised fraction	$p_{c}$, (10–30%)
Trust threshold	$T_{th}=0.5$
Initial trust range	compromised: $\left[10^{-4},0.5\right]$, normal: [0.5,1]

**Table 4:** Ablation study comparing WAA and WAY under matched scenarios. Each entry is reported as *WAA/WAY* followed by the relative change in WAY with respect to WAA. The pooled column aggregates all matched runs across n=50,100,150.

Metric	*n* = 50	*n* = 100	*n* = 150	Pooled
Number of CHs	24.918/5.516 (−77.9%)	49.914/8.013 (−83.9%)	74.769/10.094 (−86.5%)	49.867/7.874 (−84.2%)
Jain load fairness	0.771/0.914 (+18.6%)	0.760/0.896 (+17.8%)	0.746/0.884 (+18.4%)	0.759/0.898 (+18.3%)
Energy used (J)	36.370/23.708 (−34.8%)	71.405/40.183 (−43.7%)	104.269/54.125 (−48.1%)	70.681/39.339 (−44.3%)
Avg. energy per covered drone	0.727/0.474 (−34.8%)	0.714/0.402 (−43.7%)	0.695/0.361 (−48.1%)	0.712/0.412 (−42.1%)
Avg. CH trust	0.699/0.815 (+16.7%)	0.693/0.802 (+15.7%)	0.692/0.791 (+14.2%)	0.695/0.803 (+15.5%)
Path trust $T_{\mathrm{path}}$	0.699/0.815 (+16.7%)	0.693/0.802 (+15.7%)	0.692/0.791 (+14.2%)	0.695/0.803 (+15.5%)
Trust-aware link PDR	0.589/0.689 (+17.1%)	0.584/0.677 (+16.0%)	0.582/0.666 (+14.5%)	0.585/0.678 (+15.9%)
Trust-weighted total PDR	0.607/0.672 (+10.7%)	0.613/0.664 (+8.5%)	0.619/0.658 (+6.3%)	0.613/0.665 (+8.5%)

**Table 5:** WAY-CR vs. the best baseline at fixed compromised ratio (0.1) across different network sizes. Gain (%) is computed as $\frac{\mathrm{WAY}-\mathrm{Best}}{\mathrm{Best}}\times 100$ for ↑ metrics and $\frac{\mathrm{Best}-\mathrm{WAY}}{\mathrm{Best}}\times 100$ for ↓ metrics.

Trust and Delivery (higher is better)			
**Metric**	** n = 50 **	** n = 100 **	** n = 150 **
PDR_global_trust(↑)	0.6823 vs. 0.6335 (AAFSA), +7.70%	0.6758 vs. 0.6293 (TMFCS), +7.39%	0.6723 vs. 0.6391 (EESCRA), +5.19%
PDR_links_trust(↑)	0.6982 vs. 0.6547 (EESCRA), +6.64%	0.6875 vs. 0.6808 (EESCRA), +0.98%	0.6814 vs. 0.7002 (EESCRA), −2.68%
avg_ch_trust(↑)	0.8260 vs. 0.7770 (EESCRA), +6.31%	0.8150 vs. 0.8085 (EESCRA), +0.80%	0.8085 vs. 0.8295 (EESCRA), −2.53%
jain_load(↑)	0.9056 vs. 0.8565 (SSAFANET), +5.73%	0.9001 vs. 0.8178 (SSAFANET), +10.06%	0.8838 vs. 0.7983 (SSAFANET), +10.71%
**Efficiency and Overhead (lower is better)**			
**Metric**	** n = 50 **	** n = 100 **	** n = 150 **
AvgConsEnergy_perCovered(↓)	0.9630 vs. 1.9919 (TDLS), +51.65%	0.8184 vs. 1.6966 (TDLS), +51.76%	0.7302 vs. 1.5199 (TDLS), +51.96%
NUMBER_OF_CHS(↓)	5.60 vs. 30.45 (EESCRA), +81.61%	8.25 vs. 43.10 (EESCRA), +80.86%	10.50 vs. 51.35 (EESCRA), +79.55%
Avg_E2Edelay_CH(↓)	9.1942 vs. 9.1950 (CSBWOA), +0.0087%	9.1944 vs. 9.1950 (CSBWOA), +0.0065%	9.1946 vs. 9.1949 (SSACEER), +0.0033%

**Table 6:** Comparison of WAY-CR against the best baseline at each compromise level. Gain (%) is computed as $\frac{\mathrm{WAY}-\mathrm{Best}}{\mathrm{Best}}\times 100$ for ↑ metrics and $\frac{\mathrm{Best}-\mathrm{WAY}}{\mathrm{Best}}\times 100$ for ↓ metrics.

Metric	10% Compromised	20% Compromised	30% Compromised
PDR_global_trust(↑)	0.6758 vs. 0.6293 (TMFCS), +7.39%	0.6457 vs. 0.5925 (TMFCS), +8.99%	0.6232 vs. 0.5446 (TMFCS), +14.44%
PDR_links_trust(↑)	0.6875 vs. 0.6808 (EESCRA), +0.98%	0.6742 vs. 0.6725 (EESCRA), +0.25%	0.6702 vs. 0.6708 (EESCRA), −0.08%
AvgConsEnergy_perCovered(↓)	0.8184 vs. 1.6966 (TDLS), +51.76%	0.8364 vs. 1.7091 (TDLS), +51.06%	0.8845 vs. 1.7284 (TDLS), +48.83%
jain_load(↑)	0.9001 vs. 0.8179 (SSAFANET), +10.05%	0.9009 vs. 0.8130 (SSAFANET), +10.81%	0.8746 vs. 0.8128 (SSAFANET), +7.60%
NUMBER_OF_CHS(↓)	8.25 vs. 43.10 (EESCRA), +80.86%	7.95 vs. 42.20 (EESCRA), +81.16%	7.55 vs. 40.40 (EESCRA), +81.31%
Avg_E2Edelay_CH(↓)	9.1944 vs. 9.1950 (SSAFANET), +0.0065%	9.1943 vs. 9.1950 (SSAFANET), +0.0071%	9.1943 vs. 9.1950 (CSBWOA), +0.0082%

## Data Availability

The original contributions presented in this study are included in the article. Further inquiries can be directed to the corresponding author.
